# Machine learning quantitative OCT derived fibrosis indicators for corneal haze evaluation

**DOI:** 10.1371/journal.pone.0328320

**Published:** 2026-10-22

**Authors:** Corentin Soubeiran, Maëlle Vilbert, Benjamin Memmi, Cristina Georgeon, Vincent Borderie, Anatole Chessel, Karsten Plamann

**Affiliations:** 1 Laboratoire d’Optique et Biosciences, CNRS, INSERM, École polytechnique, Institut polytechnique de Paris, Palaiseau, France; 2 Laboratoire d’Optique Appliquée (LOA), CNRS, ENSTA, École polytechnique, Institut polytechnique de Paris, Palaiseau, France; 3 GRC 32, Transplantation et Thérapies Innovantes de la Cornée, Sorbonne Université, Centre Hospitalier National d’Ophtalmologie des Quinze-Vingts, Paris, France; No institution, UNITED KINGDOM OF GREAT BRITAIN AND NORTHERN IRELAND

## Abstract

Photorefractive Keratectomy (PRK) is a widely used laser-assisted refractive surgical technique. In some cases, it leads to temporary subepithelial fibrosis linked to visual haze, but objective and quantitative tools to monitor these symptoms are lacking. We here present a machine learning-based method for the classification of corneal images from spectral domain optical coherence tomography images recorded in vivo on standard clinical devices and use it to extract quantitative fibrosis indicators. Because of the transient nature and relative rarity of significant post-PRK haze, we trained the model on corneas presenting Fuchs’ Endothelial Corneal Dystrophy, causing similar, but permanent, fibrotic changes, and applied it to images from patients who have undergone PRK surgery. Our study shows that the model output (probability of pathological classification) combined with OCT-derived quantitative fibrosis descriptors provides an interpretable and explainable indicator of corneal healing for post-operative follow-up.

## Introduction

Photorefractive Keratectomy (PRK) is a widely performed laser-assisted surgical procedure for correcting refractive errors of the eye. While generally safe and effective, a notable complication is the development of corneal haze, a condition characterized by a loss of corneal transparency due to subepithelial fibrosis [1,2]. This haze can significantly impair visual quality, causing symptoms such as blurred vision, glare, and halos, which limit the success of the post-operative recovery [3]. The clinical management of corneal haze relies on careful monitoring of its progression and resolution over time. However, current evaluation methods are predominantly subjective, creating significant challenges for consistent and reliable patient care.

The standard clinical assessment of corneal haze is performed using slit-lamp biomicroscopy, where an ophthalmologist visually grades the severity of the haze based on rating scales [1]. This method is inherently subjective and suffers from high inter-observer and even intra-observer variability [4]. An ophthalmologist’s assessment can be influenced by factors such as their experience, the lighting conditions, and even the sequence of patients seen. To address this limitation, some quantitative techniques have been explored. Scheimpflug imaging, for instance, can provide a measure of corneal densitometry, which correlates with corneal scattering [2,4]. Some work aims to generate quantitative assement using slit-lamp photographs [5,6]. However, this technique has lower resolution than Optical Coherence Tomography (OCT) and does not provide physical parameters directly related to the tissue’s properties. Consequently, there is a pressing clinical need for an objective, quantitative, and reliable tool for the evaluation of corneal haze. Such a tool would enable clinicians to unequivocally attribute a patient’s visual complaints to haze, monitor its evolution objectively, and standardize treatment protocols.

Spectral Domain Optical Coherence Tomography (SD-OCT) is a non-invasive imaging modality that provides high-resolution, cross-sectional images of the cornea. It has become an indispensable tool in ophthalmology for diagnosing and managing a wide range of corneal pathologies. In the context of corneal haze, OCT images reveal a characteristic hyper-reflective band in the anterior stroma, just beneath the epithelium, which corresponds to the subepithelial fibrosis [7,8]. This morphological feature is a key indicator for clinicians when assessing haze. Interestingly, similar structural changes are observed in other corneal diseases, such as Fuchs’ Endothelial Corneal Dystrophy (FECD). In advanced stages, FECD can also lead to subepithelial fibrosis and stromal opacification, presenting with similar symptoms of blurred vision and glare [9]. The commonality of these structural alterations, particularly the anterior stromal fibrosis, suggests that a quantitative approach developed for one condition might be applicable to the other [8,10,11]. In our previous work, we developed algorithms for analyzing corneal transparency from SD-OCT images based on signal attenuation in the corneal stroma [12,13]. However, these methods did not specifically exploit the information contained within the subepithelial region, which is crucial for assessing both FECD-related fibrosis and post-PRK haze.

This study aims to address the lack of objective haze quantification by developing a machine learning-based method for the automatic classification of clinical OCT images using explanable quantifiers allowing the clinician to understand the decision process. Our primary objective is to classify corneas into two categories: “healthy” and “pathological” (i.e., showing signs of fibrosis). To achieve this, we leverage the morphological features of subepithelial fibrosis visible in OCT images. Given the transient nature and relative rarity of significant post-PRK haze, we adopted a strategy of training our model on a more readily available dataset of corneas with FECD, which presents with similar, but permanent, fibrotic changes. The trained model is then applied to images from post-PRK patients to evaluate corneal haze.

We hypothesize that the output of our machine learning model: the probability of an image being classified as pathological, can serve as a quantitative, interpretable, and explainable indicator of corneal fibrosis. This provides clinicians with a much-needed objective tool for monitoring corneal healing and managing patient care following PRK surgery. In this paper, we describe our method, from the extraction of quantitative fibrosis descriptors from OCT images to the training and validation of a classification model. We demonstrate how this approach can provide a reliable and measurable tool for the diagnosis and extendable follow-up of corneal haze in its transient stage.

## Materials and methods

We adopted an automatic classification approach based on a random forest classifier applied to physics-based descriptors of fibrosis extracted from OCT images, as described in the following section. This method was selected over deep-learning approaches, such as convolutional neural networks operating directly on raw images, for two reasons. The random forest classifier achieved the best classification performance among the methods tested. It has also been reported to be competitive with deep learning methods on tabular data [14]. Additionaly and unlike deep-learning, this machine learning approach relies on interpretable physical parameters rather than learned image features as it can be done in deep-learning, enabling clinicians to understand the rationale behind each classification. Such interpretability is essential for clinical adoption, as practitioners are more likely to integrate quantitative descriptors into their practice when the classification process is transparent rather than perceived as a “black box.”

### Morphological parameters of subepithelial fibrosis

Compared to a healthy cornea (Fig 1a), subepithelial fibrosis as observed in corneal haze [8,11] (Fig 1c) and some FECD cases [10] (Fig 1b) are characterized by three distinctive morphological features visible on OCT B-scans:

1. **Sub-epithelial hyperreflectivity.** Mature myofibroblasts and the disorganised deposition of type III collagen fibrils increase light back-scattering, producing a “hyperreflective” band in the Sub-epithelial / anterior stroma zone that is markedly brighter than the surrounding tissue [10,11] (Fig 1b–c).
2. **Blurred epithelium/stroma interface.** The intense scattering within the fibrotic zone reduces the contrast at the epithelial/stromal boundary, making it difficult to delineate on the OCT cross-section.
3. **Altered reflectivity depth profile, stromal transparency.** In a healthy cornea the reflectivity drops sharply beyond the epithelium, whereas in a fibrotic cornea the signal remains elevated well into the anterior stroma before decaying gradually.

**Fig 1:**
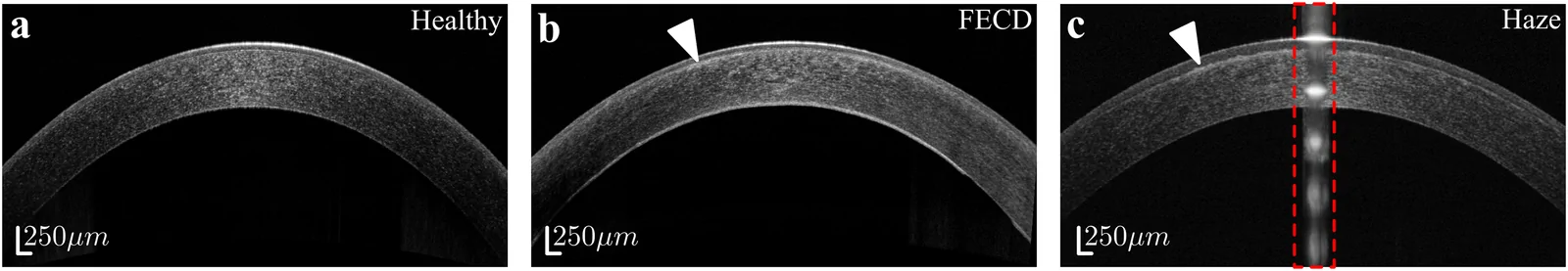
Comparison of SD-OCT images. **(a)** a normal cornea, **(b)** a cornea presenting Fuchs endothelial corneal dystrophy; the zone of subepithelial fibrosis is indicated by the white arrow, **(c)** corneal haze, the red dotted rectangle delimitates specular reflection an artefact of SD-OCT signal not to consider, the arrow indicates subepithelial fibrosis. Images have been recorded using the clinical SD-OCT RTVue-XR Avanti.

These features have a notable practical consequence. Commercial OCT devices include proprietary layer-segmentation algorithms, for instance the corneal pachymetry mapping modules of instruments such as the Optovue RTVue or Zeiss Cirrus platforms, that automatically delineate corneal layers to produce thickness maps. Because these algorithms were designed and validated on healthy corneas, they misinterpret the hyperreflective fibrotic zone as part of the epithelium: the boundary detector locks onto the deeper edge of the fibrotic band rather than the true epithelial/stromal interface. This results in a systematic overestimation of epithelial thickness in the pachymetry maps, erroneously suggesting epithelial hyperplasia when the actual cause is stromal fibrosis [9]. Although this segmentation artefact provides an indirect clinical clue to the presence of corneal haze, it also illustrates the lack of dedicated quantitative tools: existing device algorithms detect an anomaly but misattribute it, offering clinicians no direct, reliable measurement of the fibrosis itself. Building on the three morphological features described above, we designed objective, physics-based parameters that each capture one aspect of the fibrotic signature in the OCT reflectivity profile across corneal depth (the extraction procedure is detailed in the following subsections). These quantifiers serve as input features for the automatic classification model. A central design criterion was to ensure that each parameter remains physically interpretable by clinicians, so that the classification output can be traced back to observable morphological changes in the cornea.

### Standardisation of clinical images

The SD-OCT images are pre-processed following the pipeline described in full detail in [13] and available in GitHub repository [15], supplemented here with an explicit alignment step (final code available in GitHub repository [16]). For completeness and reproducibility, the eight sequential steps of the pipeline are described below. The processing pipeline allow to “flatten” the OCT images and correct artifacts, enabling the extraction of standardized depth profiles and morphological quantifiers as illustrated in Fig 2. All images have the same axial pixel size of 4.3μm, lateral pixel size varies between 5.85μm to 8.51μm depending on device recording mode (precision on device mode in “*Databases and experimental design*”). Images are considered on respective individual coordinate systems.

**Fig 2:**
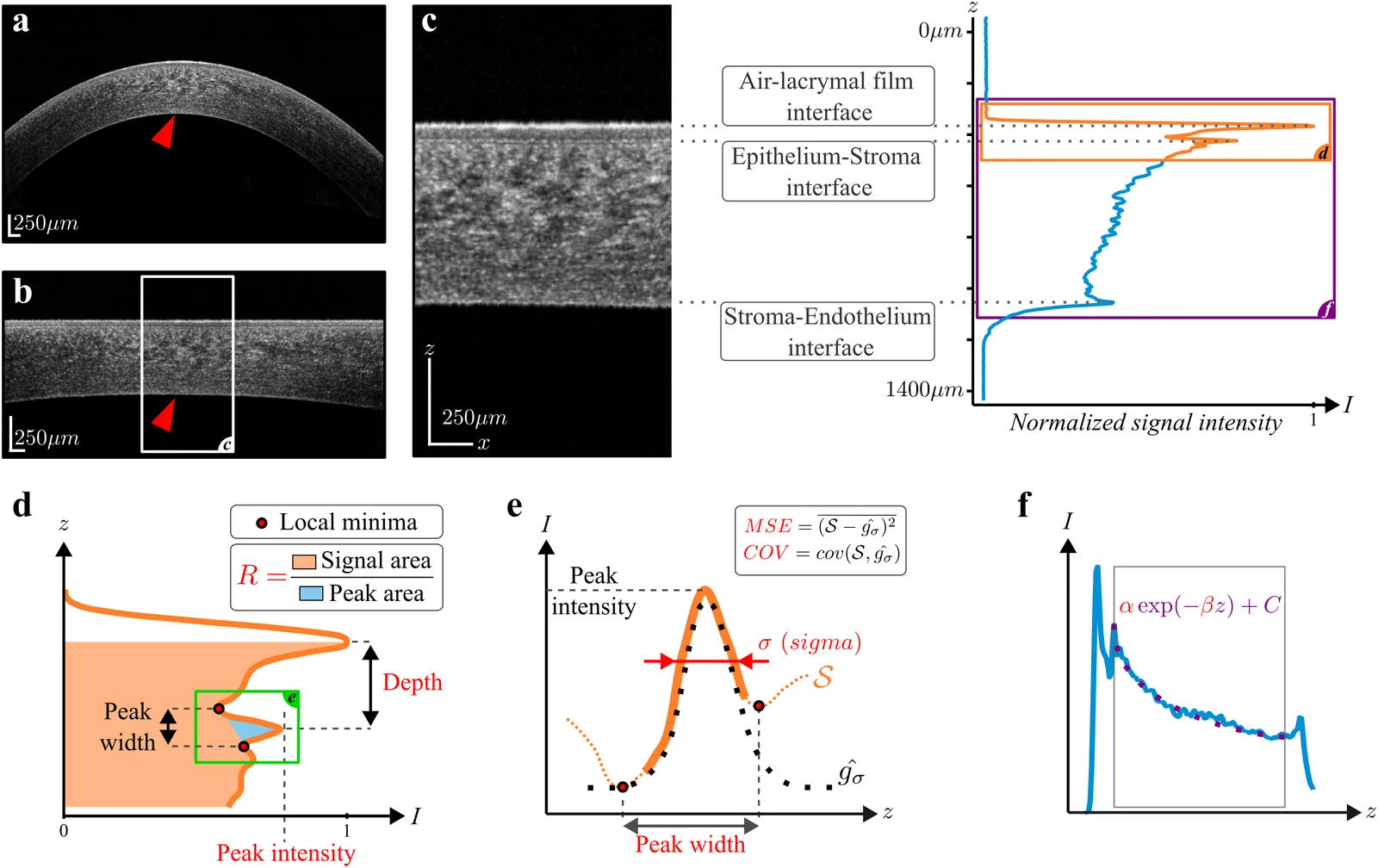
Extraction of morphological quantifiers associated with the epithelium/stroma interface performed on a healthy cornea OCT signal. **(a)**: OCT of a normal cornea acquired with clinical SD-OCT RTVue-XR. The red arrow indicate posterior stromal artefact in the central region. **(b)**: The standardized flattened OCT image, limited to the central part of 6 mm (for an overall field-of-view of 8 mm during acquisition). The red arrow indicate the same posterior region that underwent PCA correction **(c)**: 1.5 mm window and associated laterally averaged depth intensity profile. **(d)** Zoom on the two anterior intensity peaks and definition of several morphological quantifiers for the peak associated with the interface between Bowman’s layer and the corneal stroma: Depth, Peak intensity, Peak Width, and peak Area Ratio. **(e)** Additional quantifiers to this peak resulting from profile estimation: Sigma, MSE and COV. **(f)** The last two quantifiers: Alpha and Beta corresponding to stromal signal decay.

**Step A: Exposure adjustment (optional).** For over-exposed images, a histogram-based correction is applied: when the mean histogram value exceeds a threshold derived from typical clinical images, a constant offset is subtracted from all pixels to restore standard black levels in air and aqueous humour. This linear adjustment has no effect on the extracted quantifiers and is applied for visualisation purposes only, prior to any artefact detection in the case the graphical user interface is used for validation. The quantifier extraction pipeline itself operates on the raw image data, ensuring that all measurements are based on unaltered pixel values.

**Step B: Central saturation artefact detection and removal.** The column-averaged OCT intensity profile is computed and smoothed using a second-order Savitzky–Golay filter; its first derivative is then calculated. An abrupt lateral variation in the smoothed derivative identifies the specular reflection artefact at the corneal apex (illustrated in Fig 1c). Artefact boundaries are set at the lateral coordinates of the derivative extrema ±55μm, and the corresponding image columns are cropped. This cropping is necessary because the saturation artefact introduces a non-tissue-related saturated signal that would corrupt the depth-intensity profile. A graphical user interface prompts the user to validate or manually correct the automatic detection; in practice, for the large majority of good-quality clinical images, the automatic result is accepted without m**odification.**

**Step C: Anterior surface detection and A-scan alignment.** To identify the anterior corneal surface from raw image (Fig 2a) the image is pre-filtered with a 2D-Gaussian filter (σ=2 pixels) to attenuate noise and high-spatial-frequency contributions from corneal scatterers. Local maxima corresponding to the air/tear film interface are detected column by column using a robust, SNR-dependent thresholding procedure described in [13]. If detection fails at image edges due to low SNR, the anterior surface axial position is linearly interpolated from adjacent columns. The resulting axial-coordinate vector is smoothed with a median filter (window: 15 pixels). Each A-scan is then individually aligned along the depth axis so that its detected air/tear film interface coincides with a common reference depth. As this alignment is hard to manually readjust when it fails due to low SNR or pathological morphology, we implemented an automatic quality control step to what is described in [13] using normalised cross-correlation. This second alignment is performed by normalised cross-correlation [17]. In this method each A-scan is compared to the central A-scan serving as reference: the cross-correlation between the two signals exhibits a peak corresponding to their relative axial offset. Parabolic interpolation around this peak is then applied to achieve sub-pixel estimation of the shift, which is subsequently used to align the A-scans, ensuring sub-pixel co-registration along their first interface. The alignment is displayed in the graphical user interface for validation, in practice all images of our databases were correctly aligned without need for manual correction after this second step leading to flat OCT images as illustrated in Fig 2b.

**Step D: Corneal flattening and stromal region-of-interest segmentation.** All subsequent depth-intensity profiles are therefore extracted along the local axial direction of the flattened image along a vertical axis. The stromal region of interest is defined over a 6 mm wide apex-centred lateral area (reduced by the width of any artefact cropping performed in Step B). This width encompasses the optically functional central cornea and coincides with the highest-SNR region of corneal SD-OCT images [13]. This 6 mm corresponds to maximum physiological pupil dilation [18], encompasses the optically functional cornea (the central 3.2 mm being sufficient for reading vision at ±10° visual field [19]), and coincides with the highest-SNR region in corneal SD-OCT images [20].

**Steps E–F: Posterior stromal artefact correction by PCA.** The posterior stromal artefact manifests as a structured hyperreflective intensity increase within the deep central stroma, of instrumental rather than tissue origin, confirmed by its absence in SD-OCT images of a morphologically realistic corneal phantom (Rowe Technical Design Inc.; see [13]). As shown in Fig 2a (red arrow), this artefact is spatially confined to the central region of the image and exhibits a characteristic lateral signature that can be isolated from the true tissue signal. Principal Component Analysis (PCA) is applied to correct this artefact on the flatenned cornea.

- Step E: Axially, the selected region to apply PCA is limited to the stromal region, two depth (axial) margins are automatically excluded from the stromal region: +50μm beyond the epithelium/Bowman’s layer interface (high keratocyte density; non-exponential attenuation) and −30μm before the endothelium (endothelial–Descemet complex), as these zones exhibit attenuation behaviour inconsistent with the homogeneity assumption of the fitting model. These margins were set from known corneal histology and adjusted empirically to maximise measurement repeatability on normal corneas. A graphical user interface allows manual override of the segmentation when required.
- Step F: The input is a matrix of size 20×1018, where each row contains the axially-averaged OCT intensity of one of 20 equal-thickness in-depth stromal sub-layers across the 1018 lateral pixel positions; the matrix is mean-centered but not scaled. A first PCA on the full matrix identifies two dominant components(PC1 and PC2). Across a normative dataset, PC1 and PC2 accounted for approximately 94% and 4% of the variance, respectively. They respectively capture the overall depth-dependent attenuation and the curvature-driven lateral intensity decrease for PC1, and the structured lateral signature of the posterior artefact for PC2 as reported in [13]. The lateral boundaries of the artefact are derived from local minima of the PC2 reconstruction around the central image region. A second PCA is then applied to the input matrix restricted to this artefact zone; its PC1 quantifies the artefact amplitude, from which a two-dimensional correction mask with negative values is constructed and subtracted from the stromal region. Any residual intensity distortion introduced by this numerical correction is reflected in the 95% confidence interval of the output quantifiers [13].

Once applied the flattened and artefact-corrected image is illustrated in Fig 2b.

**Step G: Lateral normalisation.** The corrected image is normalised by the smoothed anterior surface intensity signal (smoothing the detected curve using second-order Savitzky-Golay filter, sliding window: 115 pixels) to compensate for the peripheral intensity loss associated with corneal curvature geometry.

**Step H: Depth-intensity profile extraction and quantifier averaging.** For each lateral position of a sliding window of width 1.5 mm across the 6 mm region of interest, the OCT intensity values are averaged laterally within the window to yield a one-dimensional depth-intensity profile. This lateral averaging reduces noise while preserving the morphological information along the surface normal. Sliding window method corresponds to incrementally (pixel-wise) slide a working window, it can mathematically be seen as a convolution by a mean operator with lateral dimension 1.5 mm. The window width of 1.5 mm was selected as a compromise between noise reduction and spatial resolution. This parameter was determined a priori and applied uniformly to all samples. In healthy corneas, where steps A–-G were performed fully automatically without manual correction, the resulting depth–intensity profiles were observed to be consistent across the 6 mm region of interest for this window width.

The nine morphological quantifiers defined in the following subsection are computed for each window position. The final value of each quantifier *Q* is obtained as a weighted mean over all window positions:


$$\begin{array}{c} \hat{Q}=\frac{\sum_{k}w_{k}\;Q_{k}}{\sum_{k}w_{k}},\;w_{k}=1-\frac{1}{2}\;\frac{\left|x_{k}\right|}{x_{\max}}, \end{array}$$ (1)

where $x_{k}$ is the lateral distance of window *k* from the corneal apex, and $x_{\max}=3$ mm is the half-width of the region of interest. The weight $w_{k}$ therefore decreases linearly from 1 at the apex to 0.5 at the periphery. This weighting reduces the influence of peripheral windows, where curvature-induced SNR degradation and residual artefacts from the flattening procedure are most pronounced, while still retaining their contribution so as not to discard clinically relevant information.

The lateral averaged intensity profile of the central region is displayed in a final graphical user interface for validation, with the display of two landmarks: the first peak corresponding to the air/tear film interface and the second peak corresponding to the epithelial basement membrane/Bowman’s layer interface. In practice it would be laborious to evaluate the landmark position across the entire 6 mm region of interest, we capitalized on the use off the weighted mean of quantifiers and the extraction of 9 different quantifiers to mitigate the impact of potential landmark detection failures in some windows.

### Definition of subepithelial fibrosis quantifiers

When examining corneal OCT images for signs of fibrosis, ophthalmologists focus on three key visual features in the anterior cornea. First, they search for hyperreflectivity in the subepithelial zone as a bright band immediately beneath the epithelium that signals the presence of activated myofibroblasts and disorganised collagen deposition [1,2]. Second, they evaluate the overall stromal transparency by assessing how dark (i.e., transparent) the stromal tissue appears on the OCT B-scan; in pathological cases, increased light scattering elevates the signal throughout the anterior stroma, reducing this darkness. Third, they examine the regularity of the anterior corneal surface, as irregular curvature may indicate prior PRK surgery and potential scarring. To objectively quantify these clinical observations, we designed physics-based descriptors that capture each aspect: peak intensity and width metrics characterize the subepithelial hyperreflective zone, exponential decay parameters quantify stromal transparency, and peak shape regularity reflects tissue homogeneity. The following quantifiers translate these visual assessments into measurable, reproducible parameters.

The morphological characterisation of subepithelial fibrosis is based on the identification of peaks of interest in the depth-averaged intensity profile of the corneal OCT signal: among others, the first peak corresponds to the air/tear film interface and the second to the epithelial basement membrane/Bowman’s layer interface (Fig 2a–c). This second peak constitutes our signal of interest because the excimer laser acts from this depth onwards for the corneal stroma remodelling, at about 100 μ m depth in the cornea. Therefore, at about this depth, the scarring reaction may lead to inflammation or even fibrosis, as shown in OCT by the deepening of the peak in question (Fig 1c).

The intensity peak associated with the epithelial basement membrane is identified via the two local minima around the peak (Fig 2c,d). It is defined by its location in depth and delimited by the distance between the axial position of the peak and the location of the two local minima (Fig 2d). A Gaussian regression is applied to the intensity profile corresponding to the peak. The Gaussian regression provides four quantifiers (Fig 2d,e):

- **Depth**: below the air/lacrimal film interface (*μ*m) to the estimated mean of the Gaussian regression. This parameter localises the primary scattering zone within the corneal stroma.
- **Sigma** (standard deviation of the peak): quantifies the spread of the peak in depth (*μ*m). In the case of fibrosis, it therefore characterizes its depth extent, reflecting how far the fibrotic reaction has propagated into the stroma from the epithelial basement membrane.
- **Peak width** (distance between the two local minima): it also characterizes the spread of the peak in depth but by looking at local minima independently of peak shape, providing a complementary metric for fibrosis thickness or shape irregularities that is less sensitive to peak asymmetry.
- **Peak intensity** (maximum intensity of the second peak compared to the maximum intensity of the first peak): it characterizes the relative intensity of the backscatter at the epithelium/stroma interface compared to the reflection at the air/lacrimal film interface. This ratio directly quantifies the hyperreflectivity observed clinically in fibrotic corneas and correlates with the density of light-scattering structures, primarily activated myofibroblasts and abnormal collagen within the anterior stroma [2].

Two parameters quantify the quality of the Gaussian regression (Fig 2e):

- **MSE** (mean square error): characterizes the deviation between the regression and the actual values of the profile.

$$\begin{array}{c} MSE=\overset{―}{{S-\hat{g_{\sigma }}}^{2}}=\frac{1}{N}\sum_{i=1}^{N}(S_{i}-\hat{g_{\sigma }}(z_{i}){)}^{2} \end{array}$$ (2)

with $S_{i}$ the actual intensity value of the profile at depth $z_{i}$, $\hat{g_{\sigma }}(z_{i})$ the corresponding value of the Gaussian regression at this depth, and *N* the number of depth points in the profile. Note that $\overset{―}{•}$ denotes the mean over all depth points.

- **COV** (covariance between the profile and the regression): quantifies the joint difference between the expectations of the profile and the Gaussian.

$$\begin{array}{c} COV=cov(S,\hat{g_{\sigma }})=\frac{1}{N}\sum_{i=1}^{N}(S_{i}-\overset{―}{S})(\hat{g_{\sigma }}(z_{i})-\overset{―}{\hat{g_{\sigma }}}) \end{array}$$ (3)

where $\overset{―}{S}$ and $\overset{―}{\hat{g_{\sigma }}}$ are the mean values of the profile and the Gaussian regression, respectively.

These last two parameters were defined following the observation of the images, and in particular, the observation that the Gaussian is sometimes poorly resolved for pathological corneas. High values of squared error or poor covariance can be a warning of pathology, as irregular peak morphology, especially asymmetry, reflects heterogeneous tissue organisation that deviates from the relatively uniform structure of healthy corneal stroma.

An exponential regression is then performed between the first peak and the endothelium to characterize the attenuation of the OCT intensity as it propagates through the stroma (Fig 2f). In healthy transparent cornea, light attenuation follows an exponential decay law due to scattering processes within the stromal tissue [12]. Deviations from this behaviour signal altered stromal transparency. Two additional quantifiers correspond to the fit variables of the regression function:


$$\begin{array}{c} f(z)=\alpha \;\exp(-\beta \;z)+C \end{array}$$ (4)

with *z* the depth in the stroma, α the initial amplitude of the signal at the anterior stroma, β the decay rate of the signal with depth, and *C* a constant representing background noise. The two quantifiers are:

- **Alpha**: the amplitude α, representing the initial scattering intensity within the anterior stroma.
- **Beta**: the rate of decay β, which quantifies how rapidly light is attenuated through the stromal depth. A lower decay rate (smaller β) indicates reduced transparency, as more light is scattered and less penetrates deeply into the tissue, a hallmark of corneal opacification [12].

Finally, a last quantifier characterizes the optical contrast:

- **Area ratio (*R*)** the ratio between the peak (epithelium/stroma interface or fibrosis) area (Fig 2d blue) and the epithelium/stroma intensity decay area (Fig 2d orange, named signal area for convinience), the bottom of the peak is generated by linear fitting between the two local minima on either side of the peak. This quantifier captures the overall contribution of the hyperreflective zone to the signal, reflecting both its intensity and extent, and thus serves as a metric of the fibrotic signature in the OCT profile.

Once these quantifiers are extracted for each image, they are used as input features for the classification model described in the next section. The interpretability of these quantifiers ensures that the model’s classification decisions can be traced back to specific morphological characteristics of the corneal tissue, facilitating clinical understanding and potential integration into practice.

The separation of quantifier distributions for a group of healthy corneas (166 OCT images) and a group of corneas with FECD (313 OCT images; inclusion criterion is selection for endothelial grafting, no stage of pathology specified) is statistically significant for seven of these quantifiers: Peak Width, Sigma, Depth, MSE, Area Ratio, Alpha and Peak intensity (S1 Fig.).

**Processing time:** The complete pipeline is implemented in Python available at [16]. Two steps involve optional graphical user interface validation: central saturation artefact detection (Step B) and stromal layer segmentation (Step D). In both cases, the automatic result is accepted without modification for the vast majority of good-quality images, and only brief training is required for non-expert users to operate the pipeline autonomously. The total processing time per image including preprocessing and quantifier extraction is on the order of 2–3 min with the graphical user interface interaction, and less than 30 seconds when the automatic results are accepted without modification on a standard workstation (Intel Core i7-8665U, 1.9 GHz, 16 GB DDR4 RAM**).**

**Robustness to low-SNR conditions and landmark detection failures**: The reliability of quantifier extraction depends on the correct identification of profile landmarks (first anterior peak, second peak, and its bounding local minima; see Fig 2). Images are acquired by professionals trained in OCT acquisition and meet established clinical quality standards. In addition to user-driven quality control via the graphical user interface for validating artefact removal and landmark detection, the pipeline integrates complementary mechanisms to preserve robustness when landmark identification is compromised by high noise levels or pathological morphologies:

- The lateral averaging over the 1.5 mm window in Step H intrinsically increases the profile SNR before landmark detection, attenuating single-column noise spikes that could trigger false minima or maxima.
- In the specific case of anterior surface detection (Step C), edge failures due to low SNR are handled by linear interpolation from reliably detected neighbouring columns.
- The Gaussian regression quality metrics (MSE and COV; see following subsection) provide per-image indicators of landmark detection confidence, and unusually high values flag potentially unreliable estimates.

### Databases and experimental design

Our primary objective is to develop an automatic classification method for clinical OCT images that can objectively identify corneal fibrosis, with the ultimate goal of quantifying post-PRK corneal haze. However, due to the transient nature of haze and the difficulty of clinical diagnosis in mild cases, insufficient validated clinical images exist to constitute a robust training database. We therefore adopted a transfer learning strategy: we trained and validated our classification model on FECD as in its advanced stages, presents similar subepithelial fibrosis localized in the anterior stroma. We subsequently applied the trained model to post-PRK patients to quantify corneal haze. This approach is justified by the morphological similarity between these conditions and the fact that FECD leads to permanent symptoms, enabling extended data collection with unambiguous clinical diagnosis.

All databases were collected between January 1, 2019, and January 1, 2023, and exported for research purposes in September 2023 (with patient or their legal guardian’s informed consent; see consent and ethical considerations section) from RTVue-XR Avanti clinical SD-OCT devices. The complete study design comprised three distinct cohorts, each serving a specific purpose (Table 1). Instrumentation and image acquisition protocols is identical with [13]: all images are exported as 8-bit grayscale B-scans with identical axial sampling (after manufacturer dewarping to physical space). The four acquisition modes reduce to two resolution groups:

- Line / CrossLine: identical sampling and geometry; 8 mm lateral FOV over 2.2 mm scan depth (4.3 μm/pixel axially; laterally 7.84 μm/pixel and 8.51 μm/pixel for Line and CrossLine, respectively), generated by the device from 30 averaged B-scans.
- Pachymetry / PachymetryWide: identical sampling between them; radial acquisitions with 6 mm and 9 mm lateral FOV, respectively, over 1.8 mm scan depth, with few averaged B-scans (exact number unknown) (4.3 μm/pixel axially; laterally 5.88 μm/pixel and 5.85 μm/pixel for Pachymetry and PachymetryWide, respectively).

**Table 1:** Databases used for the automatic classification exported from the RTVue-XR Avanti clinical SD-OCT. The pathologies of interest (corneal haze and Fuchs’ Dystrophy) have in common the development of subepithelial fibrosis in their advanced stages.

Database	Label	Patients	Eyes	Images	Modalities	Used for
Healthy Corneas	Healthy	43	83	166	Line/CrossLine (83); Pachy/PachyWide (83)	Train/Test
Fuchs Endothelial Corneal Dystrophy (FECD)	Fuchs	47	48	313	Line/CrossLine (155); Pachy/PachyWide (158)	Train/Test
Repeatability	Healthy	5	10 (5x rep.)	50	Line (50)	Test
	FECD	3	5 (4–6 rep.)	26	Line/CrossLine (26)	
Visual Haze	Haze	–	6	6	Line/CrossLine (3); Pachy/PachyWide (3)	Test
Post-Surgery Follow-up	Haze	5	9	37 (3–5 per eye)	Line (37)	Test

**Training and validation cohort (FECD model development):** This cohort consisted of 166 OCT images from healthy corneas and 313 images from patients with FECD (inclusion criterion: selection for endothelial grafting; no specific disease stage requirement). The nine morphological quantifiers described above were computed for each image. The total 479 images set has been splitted into 250 FECD, 133 healthy images for trainning and 63 FECD, 33 healthy for testing (80% train, 20% test). On the training set, we performed 5-fold cross-validation to train and select classifiers on FECD/healthy classification. Model performance metrics (accuracy, sensitivity, specificity) reported in the Results section derive from this cross-validation procedure. The test set was reserved for evaluating the final model performance on unseen data. We ensured that images from the same patient were not split between training and test sets to prevent data leakage and ensure an unbiased assessment of model generalization.

**Repeatability cohort:** To assess the reproducibility of our quantifiers and classification outputs across repeated acquisitions, we established an independent repeatability cohort comprising 10 healthy corneas and 6 corneas with FECD. These subjects underwent three consecutive OCT imaging sessions. Crucially, this cohort was entirely separate from the 479 images used for model training and validation, ensuring an unbiased assessment of measurement consistency.

**Application cohort (haze quantification):** Once the model was trained and validated on FECD data, we applied it (without any retraining or parameter adjustment) to OCT images from patients who had undergone PRK surgery and reporting visual haze symptoms. The model output for these images represents the probability of classification as pathological (i.e., presenting fibrosis similar to FECD).

**Extension cohort (post-PRK follow-up):** To further evaluate the clinical utility of our quantification method, we conducted a longitudinal follow-up of post-PRK patients. This extension cohort included 9 patients eyes with serial OCT imaging at multiple time points post-surgery. This to study the transient evolution of post surgical inflammation and fibrosis, and to evaluate how our quantifiers track the progression and resolution of corneal haze and fibrosis indicators over time, providing insights into the healing process and the potential for our quantifiers to monitor disease progression and resolution in a clinical setting.

### Interpretability

As our classification method relies on physics-based parameters derived from the corneal OCT intensity signal rather than on abstract learned representations, feature importance analysis provides direct insight into the decision-making process of the model. To achieve this, we employed SHapley Additive exPlanations (SHAP) [21], a widely used framework for interpreting machine-learning predictions. SHAP is model agnostic, meaning it can be applied to any machine learning model regardless of its internal structure, including ensemble methods, neural networks, and linear models. SHAP quantifies the contribution of each input feature to a model’s output by computing Shapley values, a concept from cooperative game theory. These values represent the marginal contribution of a feature to the prediction when considered in all possible subsets of features. For a given feature i, its Shapley value $\phi_{i}$ is computed as (adapted from [21]):


$$\begin{array}{c} \phi_{i}=\sum_{S\subseteq F⧵\{i\}}\frac{|S|!(|N|-|S|-1)!}{|N|!}[f(S\cup \{i\})-f(S)] \end{array}$$ (5)

where *F* is the set of all features, *S* is a subset of features excluding *i*, and *f*(*S*) is the model’s output based on *S*. In practice, SHAP approximates *f*(*S*) and f(S∪{i}) without retraining the model for every subset. Instead, it marginalizes the model’s predictions over the distribution of missing features. For tree-based models, the TreeExplainer variant computes these values efficiently by leveraging the tree structure to conditionally traverse decision paths and split probabilities. This approach ensures a fair allocation of the predicted outcome among all features, satisfying properties of consistency and additivity. SHAP provides both global interpretability by aggregating feature contributions across the dataset and local interpretability by explaining individual predictions. These capabilities allowed us to identify the most influential features driving the model’s decisions while ensuring transparency and alignment with the underlying physical phenomena.

## Results

### Training and validation (FECD model development)

We adopted a supervised two-class framework. The combined dataset of 166 healthy and 313 FECD images was split into a training set (80%, preserving class proportions) and a held-out test set (20%), ensuring that images from the same patient did not appear in both sets [22,23]. The nine morphological quantifiers described above serve as input features; the model output is the predicted probability of FECD classification, *p*_FECD_, which quantifies the degree of confidence in the classification.

Model selection was performed by 5-fold cross-validation on the training set: at each iteration, the model was trained on four folds and evaluated on the remaining fold. This procedure provides a robust estimate of generalisation performance while mitigating overfitting to any single data partition. We evaluated: linear SVM, radial-basis-function SVM, logistic regression, decision tree, random forest, and multilayer perceptron. The random forest achieved the highest cross-validation accuracy and was therefore retained for final evaluation on the test set (S1 Table.). As the training set is unbalanced, we used a class weight training for all thoses methods by adjusting class weights inversely proportional to class frequencies in the input data. This approach helps to mitigate bias towards the majority class and improve performance on the minority class [22]. Hyperparameters were optimized using grid search. The selected random forest model consists of 100 trees, with a minimum of 2 samples required to split an internal node (maximum depth selected according to this sample split). The Gini impurity criterion was used for measuring the quality of splits. The models were implemented using the scikit-learn library in Python [24]. Model selection was performed using 5-fold stratified cross-validation on the training set, based on the weighted F1-score, to compare candidate classifiers. The random forest model consistently achieved the best performance and was therefore selected. Following this selection, the final model was re-instantiated and retrained on the full training dataset using the chosen hyperparameters.

On the held-out test set, the random forest classifier attained a sensitivity (true positive rate) of 96.8% and a specificity (true negative rate) of 97.0%. The area under curve of the receiver operating characteristic curve (ROC AUC) is 0.9976 (Fig 3a–c). Only three images were misclassified; the ambiguous nature of these cases is examined in the Discussion. The distribution of *p*_FECD_ across the test set comprising 33 healthy, 63 FECD, and 6 haze images, is shown in Fig 3a and reveals a clear separation in the x-axis (FECD probability) between the control and pathological groups (y-axis being the model-estimated probability of FECD represented by the violin curve for data distribution visualization), underscoring the discriminative power of the physics-based quantifiers and motivating a closer examination of the few borderline cases.

**Fig 3:**
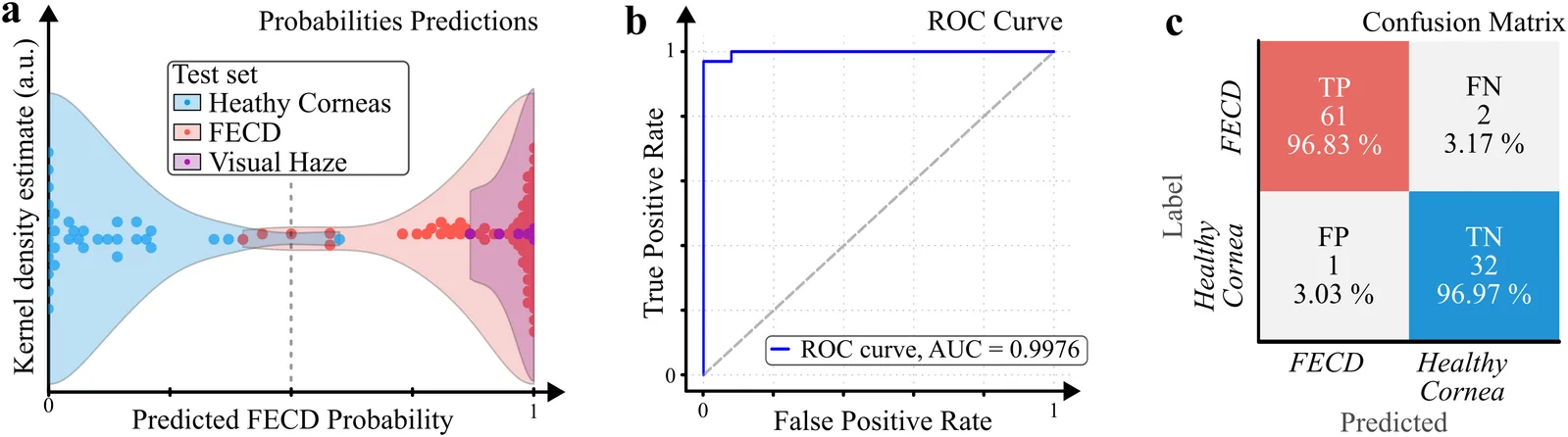
Random Forest Results. **(a)** Distribution of predicted FECD probabilities (*p*_FECD_) for healthy corneas, FECD, and visual-haze images in the independent test set. The horizontal axis corresponds to the model-estimated probability of FECD represented by the violin curve. Healthy samples cluster near 0, whereas FECD and visual-haze samples cluster near 1 (healthy mean predicted probability 10.0% std: 13.2%; Fuchs’ Dystrophy mean predicted probability: 90.9% std: 14.2%; Haze mean predicted probability 96.0% std: 4.7%). **(b)** Receiver Operating Characteristic (ROC) Curve, Area Under the Curve (AUC) 0.9976. **(c)** Confusion matrix on test set.

### Repeatability results

A repeatability study was performed to test the robustness of the classification. We tested the model on a series of 5 images acquired on the same day by the same orthoptist on the same patients, including 10 healthy subjects (10 corneas × 5 repetitions) and 3 patients with Fuchs’ Dystrophy (5 corneas × 5 repetitions). The results of the repeatability study are shown in Fig 4. The classification accuracy of the model is 100%, with a compact and statistically significant separation between the healthy and pathological groups. However, we denote a strong dispersion of results in the Healthy group (Fig 4a.) that do not correlate with the SNR index (Eq 6), nor with quantifiers dispersion as illustrated with peak width in Fig 4b suggesting that factors other than signal quality may contribute to the observed variability in the healthy group. Nevertheless, we noticed variation in the OCT signal itself in terms of signal intensity, texture or artefacts, as illustrated in S2 Fig. potentially coming from the OCT system recording process.


$$\begin{array}{c} SNR=10\log_{10}\left(\frac{P_{signal}}{P_{noise}}\right) \end{array}$$ (6)

where *P*_signal_ is the power of the signal (mean intensity of the corneal region from the air/lacrymal filp to the 1.5 mm deep, as it is the region where quantifiers are calculated) and *P*_noise_ is the power of the noise (standard deviation of the background region above and below the cornea). The SNR values for the images in the repeatability study ranged from 15 to 25 dB.

**Fig 4:**
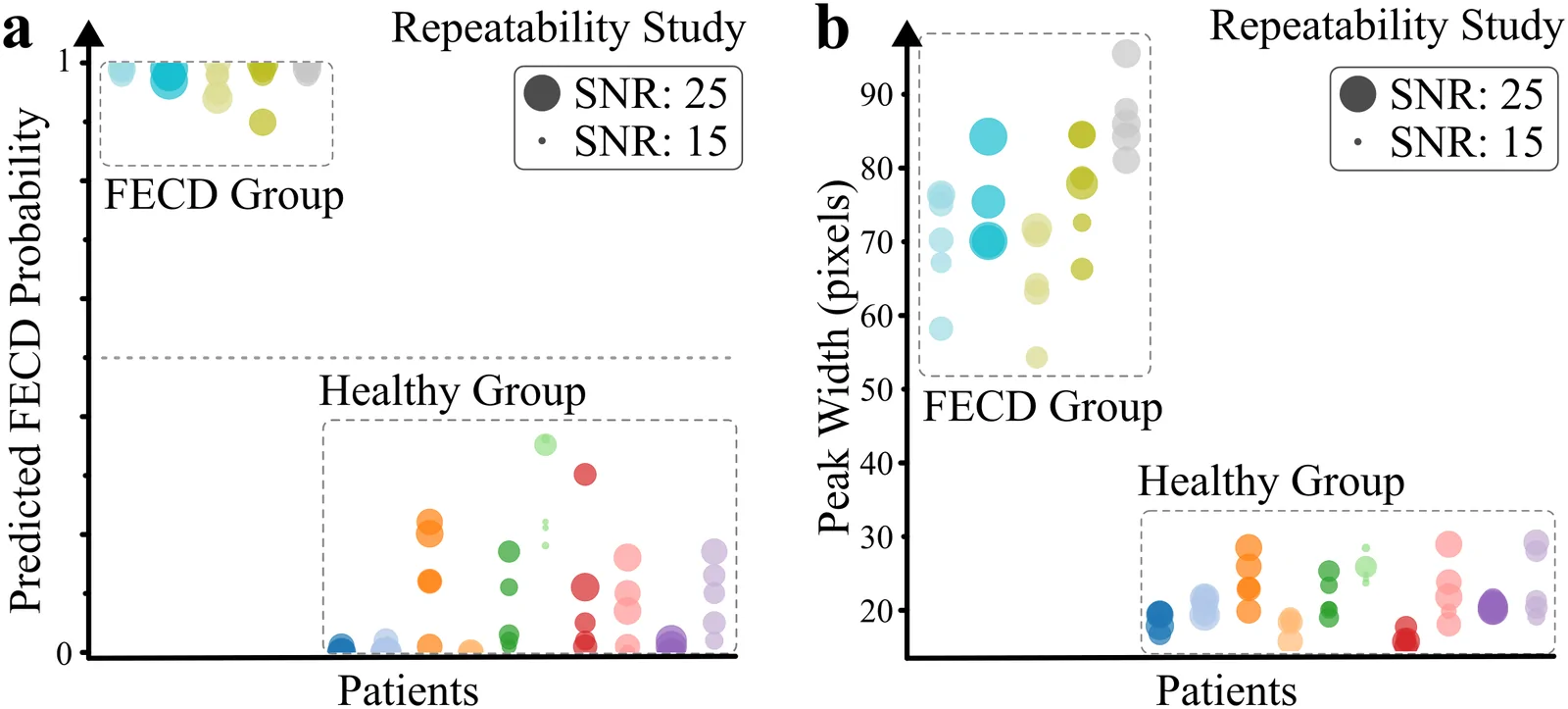
Repeatability results: study on 10 normal corneas (group control) and 5 corneas with Fuchs’ dystrophy (pathological group). **(a)** Probabilities of dystrophy classification for the 5 images per corneas, respectively normal and pathological. **(b)** Repeatability of peak width quantifier.

### Transfer on the application cohort (haze quantification)

The trained model was applied to the 6 images from patients that underwent PRK surgery and reporting vizual haze (application cohort from Table 1). The predicted probabilities of FECD classification, *p*_FECD_, for these images are shown in Fig 3a (purple). Notably, all 6 haze images were assigned a high probability of being classified as pathological, with *p*_FECD_ values ranging from 0.95 to 0.99, indicating that the model successfully identified the fibrotic features in these post-PRK corneas. This result supports our hypothesis that the morphological quantifiers derived from OCT images can serve as a reliable and interpretable indicator of corneal fibrosis, even when the model is trained on a different but related pathology (i.e., FECD). The successful transfer of the model to the haze cohort demonstrates its potential utility for objective quantification of corneal haze in clinical practice.

### Extension to post-PRK surgery follow-up

To further evaluate the model’s applicability for monitoring corneal haze progression, we applied it to a longitudinal dataset of 9 corneas (from 5 patients) who underwent PRK surgery and were followed up at multiple time points post-operatively. The predicted probabilities of FECD classification, *p*_FECD_, were tracked over time for each patient (Fig 5a). We observed that *p*_FECD_ values were elevated in the early post-operative period, consistent with the presence of subepithelial inflammation (normal after the surgery) often associated to a visual haze symptom for the patient, similarly to fibrosis. Over subsequent follow-up visits, a gradual decrease in *p*_FECD_ was noted in most patients, reflecting a probable resolution of inflammation of fibrosis and return towards a more normal corneal state. We found it insightful to represent this follow-up relatively to the initial value of the probability and therefore represent potential improvement or degradation of such quantifier (Fig 5a). Comparing Fig 5a and Fig 5b, we can observe that patients with low initial *p*_FECD_ values tend to maintain high probabilities over time, whereas patients with higher initial *p*_FECD_ values show a more pronounced decrease, suggesting a potential correlation between the initial severity of fibrosis and the trajectory of recovery.

**Fig 5:**
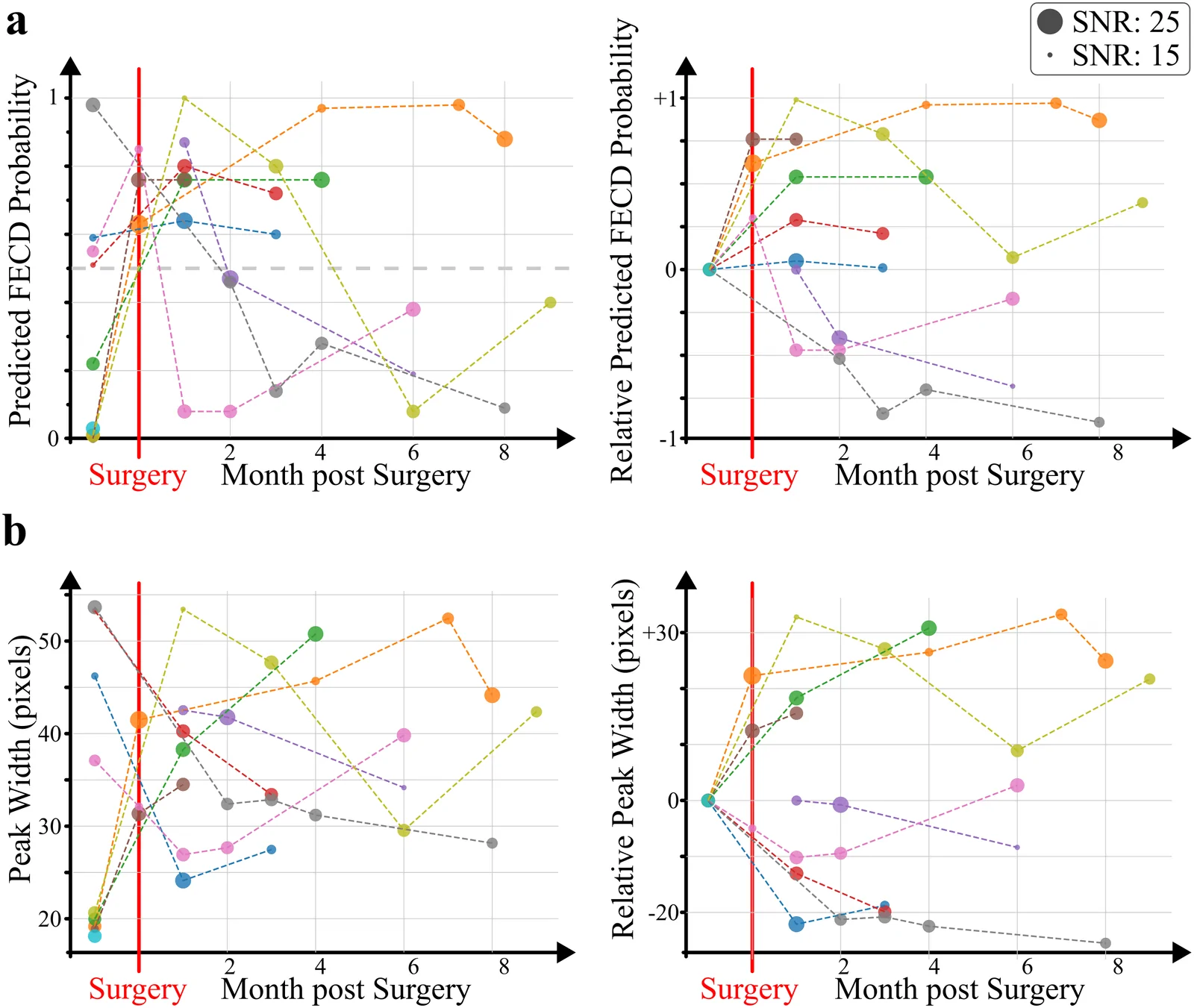
Haze post-surgery follow-up study (a) Predicted probabilities *p*_FECD_ (left) and relative to initial *p*_FECD_ probability (right) of FECD classification in Pre/Post-surgery follow-up for 9 patients. **(b)** Pre/post-surgery follow-up of peak width. The size of the points is relative to the signal-to-noise ratio of the OCT image analyzed.

Those results illustrate after the surgery a quatitative simularity of corneal structure of patient after PRK surgery with the one of patients with FECD, and therefore the potential of our model to monitor the evolution of corneal haze in a quantitative and objective manner compared to conventional clinical assessment. It suggests patient-specific trajectories of recovery that could be further investigated in larger cohorts to identify potential prognostic factors and inform personalized management strategies for patients undergoing PRK surgery.

### Interpretability using SHAP

By aggregating the absolute Shapley values across all instances, we derived a ranked feature importance profile (Fig 6a), highlighting the peak width as the most influential variable contributing to the model’s predictive accuracy. The SHAP summary plot (Fig 6a.) revealed nuanced insights into feature interactions: for instance, higher values of peak width, sigma, area ratio, and depth; and lower values of beta (reduced stromal intensity signal decay) are associated with higher SHAP values and, therefore, a higher FECD class probability prediction. Those parameters relating by construction to the 3 morphological parameters of subepithelial fibrosis: sub-epithelial hyper reflectivity (link to area ratio), blurred epithelium/stroma interface (link to peak width, sigma and depth), altered reflectivity depth profile, stromal transparency (link to beta).

**Fig 6:**
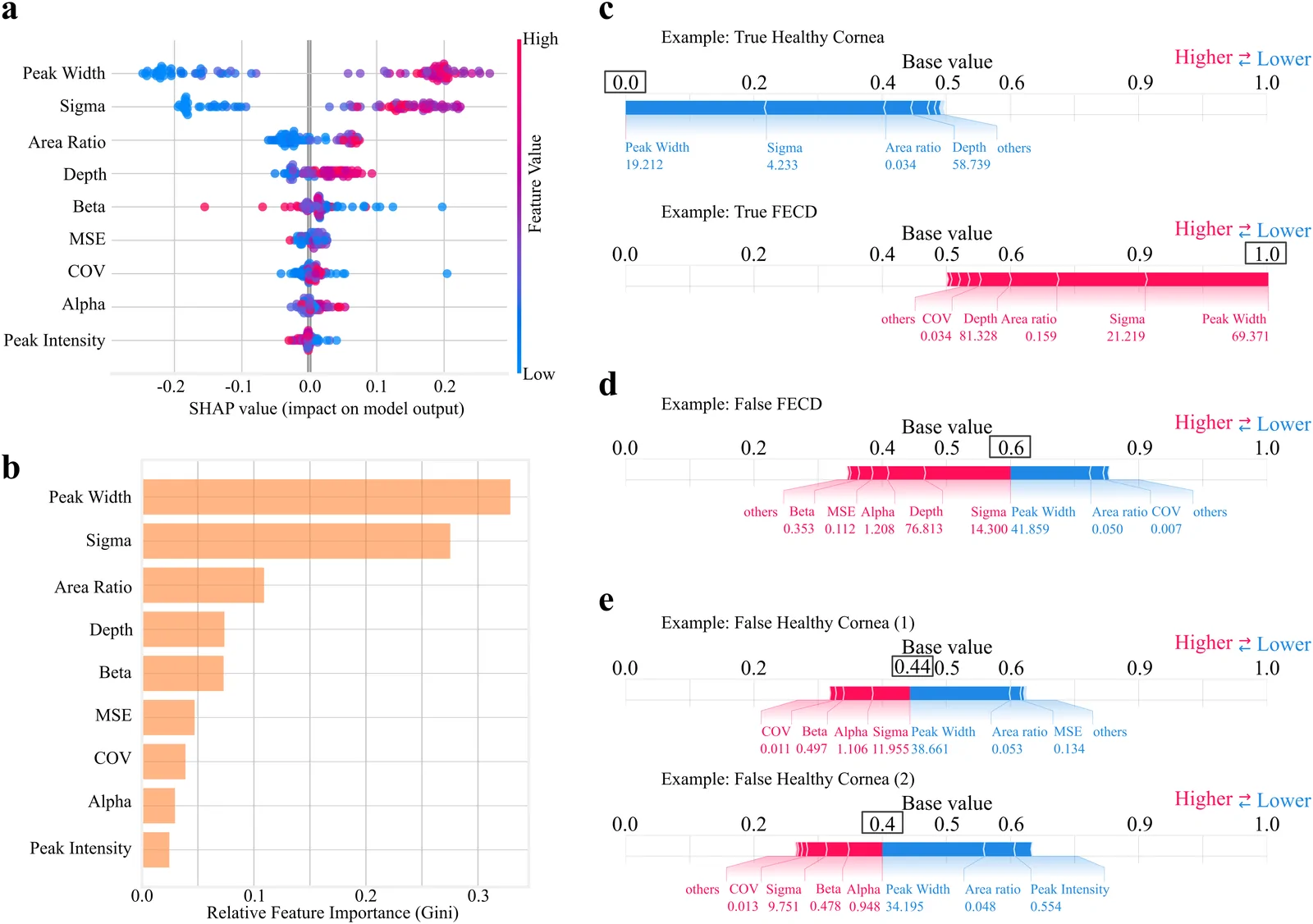
Interpretability insights (a): Shapeley Additive explanation (SHAP) features importance displaying how each training samples (dots) influences the model output through lower SHAP values (tendency to healthy class) or higher SHAP values (tendency to FECD class). Features are ordered from higher to lower impact (top to bottom). **(b)**: Gini feature importance. **(c)** Example of SHAP analysis for True Healthy Cornea (top) and True Cornea with Fuchs’ Dystrophy (FECD). The contribution of each feature to output prediction is illustrated. **(d)**: False Healthy cornea SHAP analysis. **(e)**: False Fuchs’ Dystrophy (FECD) SHAP analysis.

The feature importance deduced by SHAP is confirmed by traditional impurity-based importance scores (e.g., Gini Impurity) (a higher score indicates that the feature contributes more to the predictive power of the model) (Fig 6b.). Additionally, SHAP provides a local explanation: for each prediction, it shows how each feature either increases (pushes toward) or decreases (pushes against) the predicted value compared to a baseline (e.g., the mean prediction or expected value). For instance, on a true healthy cornea, all features participate in the predicted probability of 0 (Fig 6c, top); conversely, on a true FECD cornea, all features participate in the predicted probability of 1 (Fig 6c, bottom). In those two examples, Peak width, Sigma, and area ratio are the main participating features. Additionally, such a study can be made on misclassified data to understand what leads to a wrong prediction (Fig 6d, e) and will be discussed later. Altogether, these pieces of information indicate the peak width as a potential good biomarker of sub-epithelium fibrosis. Training the Random Forest model only with the peak width parameter leads to a classification accuracy on the FECD/healthy dataset (evaluated by cross-validation) of 88.5% (std: 2.94%), similar accuracy is found for SVM or logistic models. The use of the additional 8 quantifiers allows us to reach a testing accuracy of 96.9%.

## Discussion

### Misclassifications

Although all haze images were classified in the FECD class without retraining, one healthy cornea and two corneas with FECD were misclassified in the test set. Notably, the model outputs probabilities close to 0.5 for these three images (Fig 3a; Fig 6d,e), deviating from the rest of the dataset predictions and indicating classification uncertainty. An extended SHAP analysis of these three misclassified images reveals, for the false FECD cornea, a potential conflict between peak width and sigma quantifiers (Fig 6d). Similarly, for the false healthy corneas, a conflict between peak width, sigma, and stromal signal parameters (alpha and beta) is appearing (Fig 6e). The depth-averaged intensity profiles for these cases do not demonstrate distinctive features that would explain the misclassification (S3 Fig.), suggesting that the classification ambiguity may arise from subtle interactions between multiple quantifiers rather than from obvious morphological anomalies.

Future work could address these borderline cases by introducing additional quantitative parameters sensitive to signal density, spatial disparity, or textural information, which might help identify interpretation biases that contribute to classification errors. Alternatively, convolutional neural networks [25,26], although less interpretable than our physics-based approach, might detect local fine-scale features not captured by our depth-averaged quantifiers. Additionally, explainability techniques such as Gradient-weighted Class Activation Mapping (Grad-CAM) [27] could highlight directly on OCT images the regions participating most in the classification, potentially revealing spatial patterns that complement our quantitative descriptors.

### Post-operative follow-up after refractive surgery

According to clinical literature, transient inflammation induced haze following PRK surgery is typically expected within 2–6 weeks post-surgery, maturing between 1 and 3 months, and then regressing within a few weeks to 12 months [2,11]. Our longitudinal follow-up data are consistent with this temporal pattern: an increase in *p*_FECD_ was observed between 1 and 3 months post-operatively, followed by either a reduction (improvement) or stagnation in 8 of 9 cases, with only one case showing no improvement (Results section and Fig 5a). Fig 7 illustrates the challenge of assessing mild corneal haze: visual distinction between normal images (pre-surgery, Fig 7b, and at Month 9, Fig 7f) and corneas with haze (at Month 1, Fig 7c) is difficult, highlighting the value of the quantitative *p*_FECD_ indicator for objective post-operative monitoring. However, the sample size of this longitudinal cohort (9 corneas) is too small to draw definitive conclusions about the validity of this approach for fine-grained haze tracking over time. Extension to a larger and more temporally resolved cohort is necessary to validate the present approach. Previous densitometry studies have shown the persistence of stromal light scattering more than twenty years after surgery [28–30]: comparing our model estimates in similar long-term follow-up cases would provide valuable insight into the relationship between our physics-based quantifiers and established densitometric measures.

**Fig 7:**
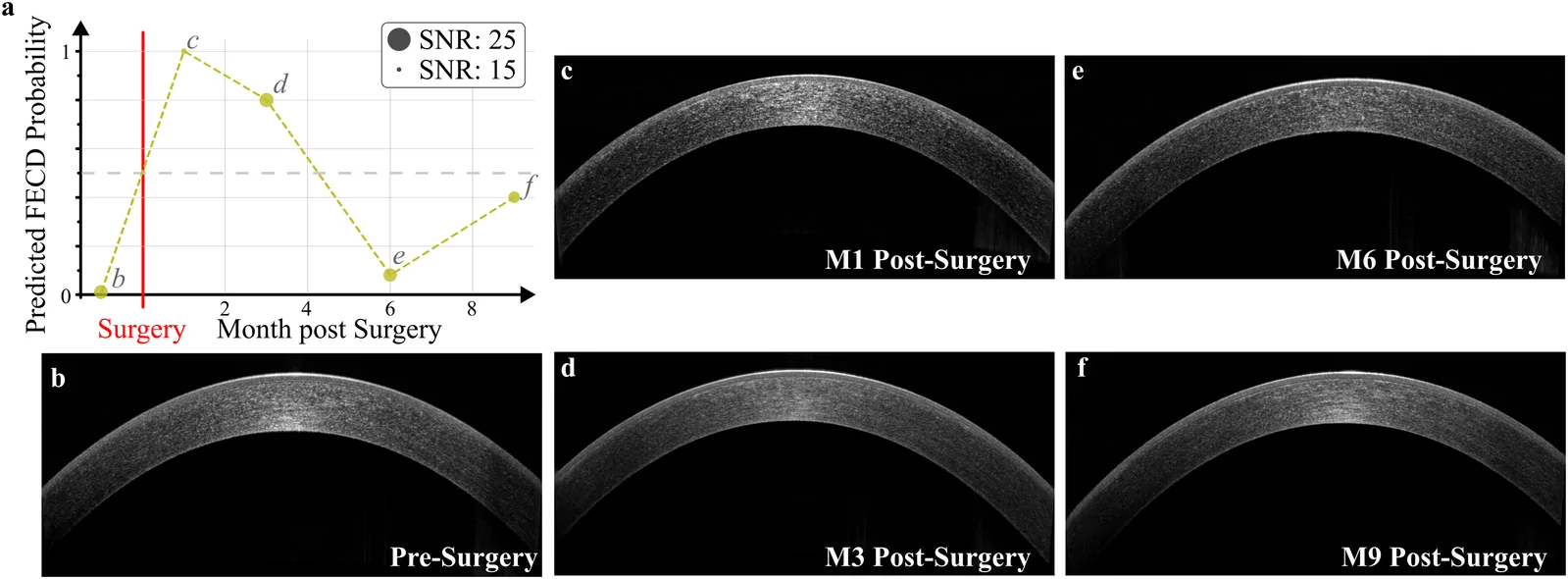
Example of surgery follow up. **(a)**: Evolution of Fuchs Dystrophy probability predicted by the model used as an indicator of fibrosis pre and post-surgery. **(b–f)** associated SD-OCT images recorded respectively before and 1,3,6,9 month after surgery.

### Clinical utility as a diagnostic aid

The primary output of our model is the predicted probability *p*_FECD_, which should be considered as one aggregated quantitative indicator among others in a clinical diagnostic aid context. The probability *p*_FECD_ reflects the degree of confidence in the classification: clear-cut values approaching 0 or 1 provide strong evidence for classification as healthy or FECD like symptoms, respectively, and can serve to confirm clinical findings obtained through conventional slit-lamp examination. Conversely, values near 0.5 or predictions that contradict expected clinical presentation should be interpreted as diagnostic warnings, prompting closer examination of the individual quantifiers and the underlying OCT images.

In such ambiguous cases, the individual physics-based quantifiers provide interpretable indicators that can guide clinical reasoning and make comparison point. For instance, the alpha and beta parameters offer information on stromal signal decay and transparency, mean square error (MSE) reflects the regularity or irregularity of the depth profile feature that are subjectively accessed in current clinical setting. SHAP analysis can help prioritise which features contributed most to a given prediction, as illustrated in Fig 6c to e. This interpretability distinguishes our approach from black-box machine-learning methods and facilitates clinical trust and adoption.

Although peak width emerged as the most influential single quantifier, achieving a cross-validation accuracy of 88.5% when used alone, this performance is insufficient for clinical deployment. While this parameter has high interpretability in terms of the axial extent of fibrosis and demonstrates strong discriminative power, a classification accuracy of 88.5% falls below the conventional 95% confidence threshold commonly required in medical statistics. By incorporating the full set of 9 quantifiers, our model attains a test set accuracy of 96.9%, exceeding this clinical acceptability threshold and confirming the added value of the multi-parameter approach for robust and reliable classification.

## Conclusion

In this paper, we propose a robust approach for automatic classification of clinical SD-OCT images for the detection of subepithelial fibrosis. The model, based on 9 morphological quantifiers and trained on FECD, achieved a test set accuracy of 96.9%. We demonstrated how training on a common disease (FECD) with permanent fibrotic features enables effective transfer learning to a rarer, transient pathology (corneal haze) presenting similar morphological characteristics. The trained model successfully classified all post-PRK corneal images reporting visual haze with high confidence(high *p*_FECD_), supporting its utility for objective haze quantification.

The primary output of our model, the predicted probability *p*_FECD_, provides a single quantitative indicator that reflects the degree of confidence in the classification and the severity of fibrotic changes. Critically, this indicator remains interpretable: through SHAP analysis, we established that it is strongly related to the axial extent of fibrosis, as captured primarily by the peak width quantifier, which guarantees clinical transparency. The physics-based foundation of our quantifiers distinguishes this approach from non-interpretable machine-learning methods and facilitates its integration into clinical practice, especially in cases where a follow-up is necessary to monitor the evolution of an inflammation, fibrosis or haze over time.

## Supporting information

S1 Fig Distribution on training set of the 9 quantifiers for each condition. Statistics are performed with Mann-Whitney U-test (no normality assumption) using Bonferonni correction of p-values (***: p-value<0.001/m; **; p-value<0.01/m; p-value<0.05/m; m = 9).
(TIFF)

S1 Table Cross-validation scores of classic machine learning classification models. (PDF)

S2 Fig Raw Repeatability data example with zoom on the apex region. **(a)** healthy cornea example, **(b)** FECD cornea example.
(TIFF)

S3 FigThe 3 Misclassified corneas and corresponding SD-OCT raw signal (right top) associated standardized signal and depth profile (right) as a density map to represent signal disparities for the depth profile (one curve is on “column” or axial profile, total number of curves is 750 for a 6 mm region), μ is the mean of the curves and σ is the standard deviation of the curves.**(a)** false Fuchs dystrophy cornea. **(b–c)** false Healthy cornea.(TIFF)
