# Supplementary material for: Machine learning quantitative OCT derived fibrosis indicators for corneal haze evaluation
Source: PLoS One. 2026 Oct 22;21(10):e0328320. doi: 10.1371/journal.pone.0328320 (PMC13690194; doi:10.1371/journal.pone.0328320)
Supplement: Supplementary file 1 [file pone.0328320.s002.pdf]

**Model**	**Cross Validation F1 score (5 folds)**	
	**Mean Score**	**Score Standard Deviation**
Random Forest	93.2%	3.61%
SVM (Linear)	91.2%	2.27%
SVM (RBF)	90.1%	3.14%
Logistic Regression	89.9%	2.20%
Neural Network	89.5%	3.55%
Decision Tree	89.0%	1.96%
