# Supplementary material for: Machine learning quantitative OCT derived fibrosis indicators for corneal haze evaluation
Source: PLoS One. 2026 Oct 22;21(10):e0328320. doi: 10.1371/journal.pone.0328320 (PMC13690194; doi:10.1371/journal.pone.0328320)
Supplement: Supplementary file 2 [file pone.0328320.s006.docx]

## Reviewers' comments:

### Response to Reviewer and Editor Comments

We sincerely thank the Editor and both Reviewers for their detailed, constructive comments. We have carefully revised the manuscript in response to all points raised. Below, we provide a point-by-point response. All changes are indicated in the revised manuscript; revised line numbers refer to the resubmitted version.

### Review Comments to the Author and Answers

**Reviewer #1:** This manuscript deals with an important clinical issue. It proposes hand-crafted features extracted from OCT images and uses classical deep learning techniques to identify clinically abnormal corneal conditions. However, serious issues exist, which need to be addressed before reconsidering for publication.

1. **Comment:** One major issue of this manuscript is the lack of clarity about the aim and the objective. The title is very unclear as “machine learning for PRK follow up”.
   In Line 149 - 150 it is stated “Our final goal is an automatic classification of clinical OCT images into two categories: "healthy corneas" and "corneal haze"”. This statement, if it holds, it should be made very early in the manuscript.
   In the abstract it is stated “we trained the model on corneas presenting Fuchs’ dystrophy, causing similar, but permanent, fibrosis symptoms, and applied it to images from patients who have undergone PRK surgery”. Immediately after that is follows “… the model output (probability of Fuchs’ dystrophy classification) …”
   Then, results are presented using 5-cross validation on the FECD images. In Table 1 several databases are mentioned without explaining the training set and the test set from which results are presented.
   Moreover, in Fig. 5 repeatability results are presented from 10 healthy and 5 Fuchs retinas, without explaining if these are different from the 166 healthy, 313 Fuch retinas, or the remaining Haze retinas of Table 5.
   Thus it is unclear to the reader what is the aim of the work.
   The authors should modify the title and rewrite the abstract to make it more specific. Following the introduction, a short subsection entitled “Objective and contributions” (or similar) should be added describing exactly that, without any ambiguity. Table 2 should be redesigned to clarify all the above.
   a. **Answer:** We understand that the initial version of the manuscript did not sufficiently clarify the aim, workflow, and dataset usage, which could lead to confusion regarding the objective and evaluation strategy. In the revised manuscript, we clarified the objective: The title has been refined to better reflect the scope of the study: derive fibrosis indicators. We believe we now explicitly state that the primary goal is the automatic classification of corneal OCT images into healthy vs. fibrotic (haze-like) corneas, using physics-based quantifiers and a machine learning model. We explain the rationale of training on FECD images (as a proxy for fibrosis) and applying the model to PRK follow-up data.
   b. In details, we added in the abstract a precision about the transient nature of the fibrosis and the link with Fuchs’ Dystrophy (and shared symptoms), and clarified the use of OCT-derived quantifiers. As the reviewer reported confusion and a structural problem in the introduction. We performed a major revision of the introduction. We now start by the source of the corneal Haze the PKR surgery, describing its clinical symptoms and presenting the current means of evaluation (revision l1-24). We explain why OCT may be more reliable for quantitative assessment (revision l26-29). We then describe the transient nature of the fibrosis occurring in Haze and why Fuch’s Dystrophy is interesting, as it shares similar symptoms but is permanent and therefore suitable to create a bigger database to access such symptoms (revision l30-42). We conclude the introduction by a clear statement of our aim (address the lack of objective haze quantification) and hypothesis. To remove ambiguity, we introduced a dedicated paragraph at the end of the Introduction (revision l43–53) explicitly stating the objective and hypothesis of the study. This paragraph clearly distinguishes: the training/validation task (classification of FECD vs. healthy corneas), from the application task (transfer to PRK haze assessment).
   c. We also clarified the use of datasets throughout the manuscript. In the revised *Materials and Methods* and *Results* sections, we explicitly describe: the composition of each dataset (healthy, FECD, PRK), the role of each dataset (training, validation, independent testing), and the cross-validation strategy (revision l376–383, Table 1 updated accordingly). By defining each cohort successively we explain what they address (revision l372-404) and kept the same order in results (revision l332-516).
   d. Regarding the confusion on specific figures: For Fig. 5 (repeatability), we now explicitly state that the subset “10 healthy subjects (10 corneas × 5 repetitions) and 3 patients with Fuchs’ Dystrophy (5 corneas × 5 repetitions)” (revision l472-473), we believe the the new structuration explicit the independent subset used exclusively for repeatability analysis, distinct from the training and validation cohorts. We clarified these distinctions in the figure caption and corresponding text, and Table 1 (and related tables) has been redesigned to clearly indicate dataset splits, sample sizes, and their respective roles (training, validation, testing,), ensuring full transparency and reproducibility.
   e. We understand that this comment was broader then the listed points so we added other modifications such as clarifying the processing pipeline (Steps A-H), the quantifiers construction (and their link to clinical insight), precised the processing time (l327-335), robustness (l337-354).
2. **Comment:** Fig. 4a is completely unclear. The “distribution” axis appears to have no meaning. The blue and red areas are not explained. Please either describe the figure extensively in the text, explaining every aspect, or remove it from the manuscript, since in its current form it only creates questions to the readers.
   a. **Answer:** Thank you for this remark. We renamed the axis (“probability density”), and the colors are detailed in the legend.
3. **Comment:** Subsection 3.2, Lines 84 - 95 “the processing chain described in Ref. 2. This pipeline is supplemented with an additional alignment procedure relying on normalised cross-correlation”
   The authors must include the exact steps that are followed, both for [2], as well as for the alignment procedure and the rest of the steps, since the above are non-trivial image processing tasks. If this is too technical for the journal, then some form of additional material should be used. In its current form, the statement is not acceptable for publication, since the methodology is obscure and researchers cannot dublicate the procedures.
   a. **Answer:** We agree. In the revised version, we have addressed this concern by explicitly detailing the complete processing chain within the manuscript (Section Materials and Methods, revision l112–220). All steps (A to H) are now described, including preprocessing, artefact detection and removal, anterior surface detection, A-scan alignment, stromal segmentation, PCA-based artefact correction, lateral normalization, and depth-intensity profile extraction. In particular, the alignment procedure previously referenced has now been fully described (revision l150). To further improve clarity and reproducibility, we ensured that all algorithmic parameters (e.g., filter sizes, thresholds, window widths) are specified in the text, and we provide mathematical formulations where applicable. As a result, the methodology is now self-contained within the manuscript and does not rely on external references for critical steps.
4. **Comment:** Caption of Fig. 2: “red square” --> “red rectangle”
   a. **Answer:** Done
5. **Comment:** Subsection 3.3 The descriptions are very brief, without definitions and clear steps. Just to mention some confusing points:
   There is no clear description how the image in Fig. 3b is generated from Fig.3a
   The “depth-averaged profile” is calculated along the Y-axis, or along the normal vector to the corneal surface ? Please define explicitly.
   Every step of the process after definition of the profile is based on clearly identifiable landmarks of the profile (first anterior maximum, 2nd maximum, minimum before and after the 2nd maximum, etc). It seems from the manuscript that if these landmarks are not correctly detected, the remaining process will fail. The authors should discuss how will their proposed system compensate for depths with increased SNR, where the profile maybe very noisy preventing the detection of these curve landmarks.
   a. **Answer:** Thank you for this comment we therefore reworked this section 3.3, which now corresponds to “Definition of subepithelial fibrosis quantifiers” section l229, additionally to the pipeline details we added in Step A-H. The definitions of quantifiers is now clearer and we mention their meaning. Additionally, regarding the implementation itself, we added details and “Processing time” (l327). We also want to mention that the code will be available upon publication for the reader as required by PlosOne.
   b. Step C now explain the figure 2 (replacing figure 3) a to b. We understand that this remark was wider than just this point, and we therefore meticulously detailed the other steps and quantifiers.
   c. Regarding landmark detection, we addressed this point in a new paragrapher “Robustness to low-SNR conditions and landmark detection failures” l337
6. **Comment:** In Line 134 the exponential regressor has a constant term C, which is missing from Fig. 3f.
   a. **Answer:** Added
7. **Comment:** In Fig. 3e the MSE is wrong (summation over the samples is missing, division be the number of samples).
   As a general comment, mathematical quantities should be rigorously defined in the manuscript in numbered Equations, with every symbol explained. Please remove the incomplete, hard-to-read mathematical definitions from the figures.
   a. **Answer:** Thank you, the MSE was displaying the upper bar that is the mathematical symbol of the mean that includes the mentioned division. However, we agree that more mathematical formalism would clarify the manuscript, we added all the formulas, including the MSE (equation 2 l279-280) that clarify the bar notation and explexitely mention it at l282. We understand the fact that MSE and COV were hard to read in figure 2 (replacing fig3), but as we want this figure to display the 9 quantifiers, we keep it by putting it in a box and coloring the 9 quantifiers in red.
8. **Comment:** Line 110: “An interpolation is performed to improve the sampling in this area …”:
   Once again, please define clearly every aspect of the step.
   Define the calculation of “Area ratio”. The relevant lines 141-143 are completely confusing.
   a. **Answer:** We now describe better in Step H (l202-228) the average profile extraction. The area ratio is also now explicated in the figure 2 (replacing fig 3) and in the text (l309)
9. **Comment:** Subsection 4.3 The purpose of the subsection is unclear. Is there any connection with the proposed system ? Please clarify
   a. **Answer:** As now we describe the cohort and their purpose from the method section we believe this aspect has been clarified (l398-404 and l512-590 for the results). We clarified for instance with l401:“This to study the transient evolution of post surgical inflammation and fibrosis, and to evaluate how our quantifiers track the progression and resolution of corneal haze and fibrosis indicators over time, providing insights into the healing process and the potential for our quantifiers to monitor disease progression and resolution in a clinical setting.“, and l521“It suggests patient-specific trajectories of recovery that could be further investigated in larger cohorts to identify potential prognostic factors and inform personalized management strategies for patients undergoing PRK surgery.“. We believe these results have a place here since Haze is transient.
10. **Comment:** Fig. 6 Please define the calculation of SNR of the OCT image, the effect it has in the prediction. Also provide the numerical values.
    a. **Answer:** Thank you, equation 6 has been added and described l483-487, legend edited snr values scale on each figure have the same scale, but we agree it needs to be in the legend for each one.
11. **Comment:** Line 236, 260 “FD class” is the same as “FECD” class ? If yes, please replace.
    Line 236 why is “additional training” mentioned ? Has it been used or discussed somewhere else in the manuscript ?
    a. **Answer:** Thank you, FD has been edited to FECD in the revision
    b. This meant that once the model was trained on healthy/FECD corneas we presented it with Haze cornea (and not re-train it on those images) it was a methodological point. Has this was unclear, we detailed each cohort more clearly in the material and methods section, and present in the same order in the results section. For this specific point this result in exposed now in revision l488-499, and the corresponding sentence edited to “The trained model was applied to the 6 images from patients that underwent PRK surgery and reporting vizual haze (application cohort from Table 1).” At l489
12. **Comment:** Fig. S3, right column: please explain in the text how are the figures generated, from how many images and how are μ, σ calculated and from which specific images in each figure
    a. **Answer:** We included this remark in the revision by defining those parameters and detailing the image generation.

**Reviewer #2:** Soubeiran et al have presented a machine learning based approach to classify healthy corneas vs those with Fuchs’ dystrophy. The study setup seems appropriate and the results are promising.
However, as a general PLOS One reader unfamiliar with this medical field, I was unable to fully follow the manuscript and found the description of both the disease and the methodology lacking for a comprehensive review. I have described both my major and minor concerns below:

1. **Comment:** As PLOS One caters to a diverse audience, it would be helpful if the authors presented more information about the medical condition and the problem motivation. I appreciate the need to keep the manuscript succinct, and do not expect a full background of every medical term. However using simpler language, describing the most relevant terms, and emphasizing the motivation of this study would make the manuscript more broadly accessible.
   a. **Answer:** We reshaped the introduction to introduce more medical background (revision l1-24), we refined the relevance of OCT (revisionl26-42) and clarified the aim (revision l43-53) to make the revised manuscript more accessible in accordance with this comment.
2. **Comment:** The methodologies are not described sufficiently for a comprehensive review. For example, data cleaning methodologies are only subjectively described. Feature ranges are missing. Image labeling methodology is missing. Hyperparameter selection is missing.
   a. **Answer:** Thank you for those remarks. Overall we tried to clarify in the revision each step of the process to answer this concern. Specifically to those mentioned points: we now give a description of the methodology for the pipeline (Steps A to H, revision l118-220) we specified range values of algorithm varible; the clinical parameters used for labelling are now described (revision l76-90); hyperparameter selection precised in revision l455-459; we detail the clinical meaning of our quantifiers, their equation, range of the quantifiers are available in S1 fig; in to answer this concern.
   b. We also understand that this point was more global than just mentioned point; this is we rewrote different sections. The introduction is to make our objective clearer and give more context on the clinical assessment, the automatic approach of the literature, and the missing parts. We reworked the material and methods section to clarify the methodology and the datasets. Specifically, we also detailed more extensively each cohort so the results section is more reproducible.
3. **Comment:** Line 15 - Suggest including the name of the machine learning algorithm in the title, abstract, and Introduction. ML is too broad a term to describe the authors’ specific work.
   a. **Answer:** We refined the title while keeping “machine learning”, although machine learning is indeed a broad term, it still refers to a specific type of algorithm that we specifically tested in this paper and referenced both in the manuscript text (revision l448-450) and in S1 Table. However, to add some nuance on the machine learning vs. deep learning in revision l70-71.
4. **Comment:** Line 28 - It is not clear why the authors have started the Introduction with a reference to their own previous work. While both works relate to Fuchs, the previous work has not included any ML. I would suggest starting the Introduction with the specific problem being solved in this work, and referencing previous work in a later paragraph.
   a. **Answer:** We agree with this remarker and changed the introduction to start by the source of the corneal Haze the PKR surgery, described it’s clinical symptoms and presented the current means of evaluation. We explain why OCT may be more reliable for quantitative assessment. We then describe the transient nature of the fibrosis occurring in Haze and why Fuch’s Dystrophy is interesting as it shares similar symptoms but permanently and is therefore suitable to create a bigger database to access such symptoms. We conclude the introduction by a clear statement of our aim (address the lack of objective haze quantification) and hypothesis.
5. **Comment:** Introduction - There is almost no literature review in Introduction. The authors have claimed that they are not aware of quantitative tools but they have not provided sufficient data to support their statement. Additionally, even if quantitative tools are not available, surely there would be other work related to Fuchs that motivates the authors’ work and highlights why haze diagnosis is required. Other work will also serve as a comparison for how the authors’ ML model improves upon existing evaluation performance.
   a. **Answer:** Similarly, we addressed this remark by refactoring the introduction, adding a deeper literature review and clinical context.
6. **Comment:** Line 49 - What are the missing physical parameters?
   a. **Answer:** The idea was to mention that current means of evaluation are not based on any physical parameters and are referred as “presence of haze”. We can’t list what is missing but can assess that there are none. However, we re-worked this sentence by extending what the ophthalmologist is looking for (revision l14-19) and explained the difference between Slit-lamp (subjective that can’t provide “values” or physical parameter) and OCT (that provide a signal linked to the cornea absorption) (revision l19-21). Finally, we conclude on why there is a need for quantitative/objective assessment (revision l24-25).
7. **Comment:** Fig 1 - What are Ref 3 and Ref 6?
   a. **Answer:** References are now fixed in the revision (Ref3 = revision file ref [1], Ref 6 = revision file ref [2])
8. **Comment:** Line 56 - Please specify the algorithm instead of broad term like “automatic classification approach”.
   a. **Answer:** We added a mention to Random Forest in revision l64 corresponding to this sentence in the initial submission.
9. **Comment:** Line 60 - The sentence is unclear.
   a. **Answer:** We tried to clarify this in revision l70-75 corresponding to this sentence in the initial submission by detailing the difference between deeplearning extracting image features that can’t be interpreted by “human” and features created by humans (interpretable) passed to machine learning for classification.
10. **Comment:** Line 70 - The sentence is unclear.
    a. **Answer:** We added a better description of the “pipeline” in the revision and deeply explained what we are performing in Step B (revision l127-134) to make this clearer.
11. **Comment:** Line 74 - What segmentation algorithms are being referred to? If there are segmentation algorithms applied here, how does this relate to authors’ pervious statement about missing quantitative tools?
    a. **Answer:** As a big part of this paragraph was unclear for the reviewer, we re-worked it. For this specific point, we now detail in revision l92-95 that the commercial OCT device has segmentation tools that we detailed. But we mentioned their limitations and are not using them. We think that this new paragrapher from l91to 110 gives a better overview.
12. **Comment:** Line 75 - What is the issue with “overestimation of the epithelial layer thickness”?
    a. **Answer:** Similarly to the previous answer, we are now detailing l94 this point.
13. **Comment:** Line 78 - Since the “marked morphological features” are an essential input in the authors’ model, it is insufficient to refer to their description in another reference. I would suggest including a description in this manuscript.
    a. **Answer:** We now include this description in step B detailed in revision l127-137.
14. **Comment:** Line 85 - What is Ref 2?
    a. **Answer:** References are now fixed in the revision (Ref 2 = [13] in revision)
15. **Comment:** Line 85 - What is the “additional alignment procedure”?
    a. **Answer:** Now described in Step C, specifically in revision l148 to 155.
16. **Comment:** Line 87 - How much time and effort is required to detect the “central saturation artefact”? If its detection is essential to leveraging the authors’ methodology, would the image analysis already be done, thus making their approach extraneous?
    a. **Answer:** Now detailed in Step B (l128-136), concerning the time and effort we added a “Processing time” section in revision l327-334.
17. **Comment:** Section 3.2 - Would these image processing capabilities be available to physicians who intend to use the authors’ methodology?
    a. **Answer:** Code is available at publication but we now provide a detailed pipeline (Step A-H) and processing time information specifically l333.
18. **Comment:** Line 90 - Please include more description and reference for PCA.
    a. **Answer:** Yes, now in E-F l164-195.
19. **Comment:** Line 90 - How are low intensity regions defined and removed?
    a. **Answer:** Now in Step D with a precision in footnote p5.
20. **Comment:** Line 91 - How is 1.5 mm chosen?
    a. **Answer:** Now detailed in revision l203 and 207: “The window width of 1.5mm was selected as a compromise between noise reduction and spatial resolution, on healthy corneas where steps A-G steps was performed automatically without need for manual correction, we observed that the depth-intensity profiles extracted was constant across the 6~mm region of interest for this window width.”
21. **Comment:** Line 93 - What is the functional form of the weighting function? How is the weight range chosen?
    a. **Answer:** Now detailed in revision l217-219:”This weighting reduces the influence of peripheral windows, where curvature-induced SNR degradation and residual artefacts from the flattening procedure are most pronounced, while still retaining their contribution so as not to discard clinically relevant information”. We also precised the weight formula in equation 1). We also mention in l224-228: “In practice, it would be laborious to evaluate the landmark position across the entire 6mm region of interest; we capitalized on the use off the weighted mean of quantifiers and the extraction of 9 different quantifiers to mitigate the impact of potential landmark detection failures in some windows.”
22. **Comment:** Fig 3 - “peak Area Ratio” is not described in Fig 3d.
    a. **Answer:** We now include it and named it R.
23. **Comment:** Line 119 - What is being defined in item 4?
    a. **Answer:** We now start each physical parameter by its name
24. **Comment:** Line 143 - What is meant by “available simultaneously”?
    a. **Answer:** We meant that once the profile is extracted, we can extract those 9 quantifiers. But we agree it was confusing, we removed this sentence, transitioning with “Once these quantifiers are extracted for each image” in revision l316.
25. **Comment:** Line 153 - It would be helpful if the authors provided evidence about how the classification model applied to FECD images justifies the model’s application for corneal haze detection.
    a. **Answer:** Thank you for this remark. We now give a clear clinical point in revision l80 to 90 to detail what changes in case of fibrosis before detailing the quantifier and detailing for each one what they are “designed” to detect/measure in this context. Then, when presenting the FECD you are mentioning in this question, we detailed: “due to the transient nature of haze and the difficulty of clinical diagnosis in mild cases, insufficient validated clinical images exist to constitute a robust training database. We therefore adopted a transfer learning strategy: we trained and validated our classification model on FECD as in its advanced stages, presents similar subepithelial fibrosis localized in the anterior stroma.” (revision l358-362). Note also that now in the introduction (revision l35-38) we are citing 3 papers on that topic: “presenting with similar symptoms of blurred vision and glare [9]. The commonality of these structural alterations, particularly the anterior stromal fibrosis, suggests that a quantitative approach developed for one condition might be applicable to the other [8, 10, 11]”
26. **Comment:** Line 155 - Are the 9 quantifiers the features for the ML models?
    a. **Answer:** Yes we now explicitly mention it from the beginning l64 “classification approach based on a random forest classifier applied to physics-based descriptors of fibrosis extracted from OCT images, as described in the following section.“ and after detailing the quantifiers at l316 “Once these quantifiers are extracted for each image, they are used as input features for the classification model described in the next section”
27. **Comment:** Line 164 - How would the doctors label the images? Would they use any of the 9 quantifiers to make their determination?
    a. **Answer:** We now mention this explicitly in revision l80-90 by explaining what the doctor use to characterize those images. Then when detailing the quantifier, we explain their link to clinical observation (why we choose/buid them) for instance l273 “his ratio directly quantifies the hyperreflectivity observed clinically in fibrotic corneas and correlates with the density of light-scattering structures, primarily activated myofibroblasts and abnormal collagen within the anterior stroma [2]”
28. **Comment:** Line 169 - “very precisely” is a subjective term, and not descriptive in this context.
    a. **Answer:** We agree, removed
29. **Comment:** Line 170 - How did the authors train SVM, determine its hyperparameters, and experiment with reducing overfitting?
    a. **Answer:** We now describe Hyperparametrization in l455-459.
30. **Comment:** Line 172 - Does this mean that no label separation was done for implementation of SVM? How can the models performances be compared if they were trained with different datasets?
    a. **Answer:** Maybe it was unclear but a classic methodology in the literature if sometime to train SVM in a single class (as the cited reference [20]) (for instance healthy) and then present a new dataset not from the original distribution (for instance pathological corneas FECD or Haze) to detect if it fall “within” or “without” the training distribution. In our case, it did not work, and we mention it here. Has it seemed unclear, we reworked this sentence at l433: “in which only the healthy cornea class is modelled in detail and unknown images are classified according to their deviation from this reference distribution.”
31. **Comment:** Line 177 - How did the authors select 50% as the probability threshold, especially because the training dataset is unbalanced?
    a. **Answer:** We understand that the mention of the 50% was unclear, additionally in fact this information is not used in the rest of the paper as it was an arbitrary value, we removed this mention as it does not affect any result nor conclusion. We consider this probability as a continuous value that “quantifies the degree of confidence in the classification” (revision l444)
32. **Comment:** Line 184 - After cross-validation, since there will be 5 trained models, which one of the models was applied to the test set?
    a. **Answer:** We now explicitly mention this in l460 “On the held-out test set, the random forest classifier attained an accuracy “. Additionally, in Table 1 we noted the use of each set, and detailed the exact methodology in revision l376-383.
33. **Comment:** Line 186 - The sentence structure makes it seem that the high TPR and FPR at a single threshold contributes to the high ROC, which is inaccurate since ROC is generated over the entire threshold range.
    a. **Answer:** We agree it is indeed the reverse ROC taken at one value (threshold) that gives the sensitivity/specificity. We edited the sentence accordingly, removing the causality now in revision l460-463.
34. **Comment:** Line 218 - Suggest changing AI to ML to remain consistent with the rest of the manuscript.
    a. **Answer:** We strongly agree to replace this part. We also clarified why we specifically use ML in revision l64-75.
