# Supplementary material for: Machine learning quantitative OCT derived fibrosis indicators for corneal haze evaluation
Source: PLoS One. 2026 Oct 22;21(10):e0328320. doi: 10.1371/journal.pone.0328320 (PMC13690194; doi:10.1371/journal.pone.0328320)
Supplement: Supplementary file 3 [file pone.0328320.s007.docx]

**We would like to thank the reviewer for their detailed and informative comments. We addressed all comments as listed below:**

Reviewer #1:

1. Comment 1 reviewer 1:The main general comment is that the quality of all figures is far below standard, thus, many of them are hardly readable. They should be reproduced with higher dpi. Ideally they should be included in the relevant body of the text, rather than put at the end of the whole document.
   - Answer 1 (to reviewer 1): We fully agree on the importance of figure quality. The apparent low resolution is an artefact of the journal’s compilation workflow: following the editorial policy, the figures are not embedded in the manuscript file but are uploaded as individual high-resolution TIFF files (>600 dpi), which the editorial system inserts automatically at full resolution in the reviewer PDF. In the present resubmission, per the editorial office’s request, the figures have accordingly been removed from within the manuscript file, leaving only the captions in place, and are provided as separate TIFF image files. We remain happy to adjust figure placement or format according to the journal’s production requirements.
2. Comment 2 reviewer 1:Line 140: Step C: is the Gaussian 1D (for each A-scan), or 2D ? If the latter case, is the σ same for both dimensions ? (are the pixels square) ?
   - Answer 2 (to reviewer 1): 2D Gaussian is performed; lateral/axial pixel size varies from 1 to 2 depending on the recording mode of the device (Line, Pachy, Cross, and Pachywide) images; axial pixel size is consistent across modes (4.3um), all modes are considered laterally in a physical distance coordinate system (in mm). To clarify this, we added line 118 (“Standardisation of clinical images” first paragraph): “Images have the same axial pixel size of 4.3um, lateral pixel size varies between 5.85um to 8.51um depending on device recording mode. Images are considered on respective individual coordinate systems.”; We also specified the acquisition mode and its repartition in “Databases and experimental design.”; Added: “2D Gaussian”
3. Comment 3 reviewer 1:Step E. Are the two margins (50μm and 30μm) set manually ?
   - Answer 3 (to reviewer 1): Automatically validated by the user. Edited l174: “Automatically excluded”
4. Comment 4 reviewer 1:Step F: The variance of the 1st and 2nd PCA component are set to 94% and 4%, however there seems to be no reason for this to hold in the general case.
   - Answer 4 (to reviewer 1): The 94% and 4% values reported for PC1 and PC2 are not preset parameters, but empirically observed values computed by running the PCA on each individual image of the normative dataset (n = 83 normal corneas), following flattening and segmentation of the stroma into 20 equal-thickness sub-layers. The method was developed and iteratively refined on a larger set of normal corneal SD-OCT images prior to this study, providing empirical confidence in the stability of the component interpretation across normal corneas acquired with the same device and protocol. Component selection is not based on these specific percentages, but on the physical interpretation of each component – which is grounded in the known physics of the SD-OCT corneal signal. In this strongly constrained data structure (same device, same protocol, homogeneous normal stroma), PC1 captures the dominant depth-dependent attenuation trend, and PC2 captures the first structured lateral deviation from it, corresponding to the posterior stromal artifact. This interpretation was validated consistently across all images in the dataset. Indirect validation is provided by the fitting results: after correction, stromal attenuation profiles are well described by a mono-exponential decay model (Birge ratio Br < 10 for all normal corneas, IDR: 1.8–4.1), as expected for homogeneous tissue. This would not hold if the PCA-based correction introduced systematic distortions in the depth-attenuation profile. We acknowledge that individual variability around the reported variance values was not documented – these should be understood as representative values observed across the normative dataset, not as fixed algorithmic thresholds.
   - We therefore propose the edit by changing the sentence “*A first PCA on the full matrix identifies two dominant components: PC1 (94% of variance), capturing the overall depth-dependent attenuation and the curvature-driven lateral intensity decrease, and PC2 (4% of variance), isolating the structured lateral signature of the posterior artefact.*” to “*A first PCA on the full matrix identifies two dominant components(PC1 and PC2). Across a normative dataset, PC1 and PC2 accounted for approximately 94% and 4% of the variance, respectively. They respectively capture the overall depth-dependent attenuation and the curvature-driven lateral intensity decrease for PC1, and the structured lateral signature of the posterior artefact for PC2 as reported in […].”*
5. Comment 5 reviewer 1:Step G: Is the filtering performed in 1D ?
   - Answer 5 (to reviewer 1): We are smoothing a curve corresponding to the anterior surface intensity signal. To clarify this: edited l198 to “The corrected image is normalised by the smoothed anterior surface intensity signal (smoothing the detected curve using second-order Savitzky-Golay filter, sliding window: 115 pixels) to compensate for the peripheral intensity loss associated with corneal curvature geometry.”
6. Comment 6 reviewer 1: Step H: is the 1.5mm-width window placed in a overlapping manner, or is it applied 4 times to cover the 6mm width ?
   - Answer 6 (to reviewer 1): The 1.5mm-width window is in indeed applied in an overlapping manner (“moving window”), to clarify this we added a footnote for “sliding window” : “sliding window method corresponds to incrementally (pixel-wise) slide a working window, it can mathematically be seen as a convolution by a mean operator with lateral dimension 1.5mm.”
7. Comment 7 reviewer 1: Fig.3(a) needs to be explained in the text, because the authors draw important conclusions from it, about the quality of the classifier. The shape is not self-explanatory, the Y axis label is still confusing (does not remind of any probability density function) and the different colors that seem to contain are not visible. Its title is also too general.
   - Answer 7 (to reviewer 1): the Y-axis often represents kernel density estimate (KDE) width, sometimes called “density” or “Probability density”. We propose to edit panel a legend from : “distribution of predicted fibrosis probabilities ($p_{\mathrm{FECD}}$) (Healthy mean predicted probability 10.0\% std: 13.2\%; Fuchs' Dystrophy mean predicted probability: 90.9\% std: 14.2\%; Haze mean predicted probability 96.0\% std: 4.7\%).” To “Distribution of predicted FECD probabilities ($p_{\mathrm{FECD}}$) for healthy corneas, FECD, and visual-haze images in the independent test set. The horizontal axis corresponds to the model-estimated probability of FECD represented by the violin curve. Healthy samples cluster near 0, whereas FECD and visual-haze samples cluster near 1 (healthy mean predicted probability 10.0\% std: 13.2\%; Fuchs' Dystrophy mean predicted probability: 90.9\% std: 14.2\%; Haze mean predicted probability 96.0\% std: 4.7\%).”
   - Additionally in the text we edited “reveals a clear separation between the control and pathological groups” to “reveals a clear separation in the x-axis (FECD probability) between the control and pathological groups (y-axis being the model-estimated probability of FECD represented by the violin curve for data distribution visualization).”
   - We renamed the y-axis to “Kernel density estimate (a.u.)” for clarity
8. Comment 8 reviewer 1: Line 341-342 needs rewriting
   - Answer 8 (to reviewer 1): Text was: “In addition to user quality control of the automatic detection of the artefact removal and these landmarks via the graphical user interface, the pipeline incorporates several complementary mechanisms to maintain robustness when these landmarks are difficult to detect due to elevated noise or pathological morphology”
   - Proposed version: “In addition to user-driven quality control via the graphical user interface for validating artefact removal and landmark detection, the pipeline integrates complementary mechanisms to preserve robustness when landmark identification is compromised by high noise levels or pathological morphologies:”
9. Comment 9 reviewer 1: In Fig. 5b the relevant probability spans [-1,1], whereas in (a) the range is [0,1]
   - Answer 9 (to reviewer 1): Indeed, right panel represents the value relative to the pre-surgery value, as left panel span initial values from 0 to 1, relatively to initial value it can span from -1 to 1. In the figure caption “Predicted probabilities *p*FECD (left) and relative to initial *p*FECD probability (right) of FECD classification in Pre/Post-surgery follow-up for 9 patients.”
   - It was also precised line 510 “We found it insightful to represent this follow-up relatively to the initial value of the probability and therefore represent potential improvement or degradation of such quantifier” and line 512 “Comparing Fig 5a and Fig 5b, we can observe that patients with low initial *p*FECD values tend to maintain high probabilities over time, whereas patients with higher initial *p*FECD values show a more pronounced decrease, suggesting a potential correlation between the initial severity of fibrosis and the trajectory of recovery.”

Reviewer #2:

1. Comment 1 reviewer 2: Line 69: Sentence structure
   - Answer 1 (to reviewer 2): Sentence was : “It is also repported as a competitive compare to deep learning on tabular data” we edit it to “It has also been reported to be competitive with deep learning methods on tabular data”
2. Comment 2 reviewer 2: Line 70: *Additionally*:
   - Answer 2 (to reviewer 2): corrected thank you
3. Comment 3 reviewer 2: Line 70, 72: *deep-learning*.
   - Answer 3 (to reviewer 2): corrected thank you
4. Comment 4 reviewer 2: Line 152: Can the authors include a summary of how their normalized cross-correlation method enables a second re-alignment?
   - Answer 4 (to reviewer 2): Yes, we propose to complete this sentence by: “This second alignment is performed by normalised cross-correlation [17]. **Each A-scan is compared to the central A-scan serving as reference: the cross-correlation between the two signals exhibits a peak corresponding to their relative axial offset. Parabolic interpolation around this peak is then applied to achieve sub-pixel estimation of the shift, which is subsequently used to align the A-scans, ensuring sub-pixel co-registration along their first interface.**”
5. Comment 5 reviewer 2: Line 154: *alignment*:
   - Answer 5 (to reviewer 2): corrected thank you
6. Comment 6 reviewer 2: Line 184: *mean-centered*:
   - Answer 6 (to reviewer 2): corrected thank you
7. Comment 7 reviewer 2: Line 208: The statement indicates that the steps A-G would be applied differently to healthy and unhealthy corneas. This could introduce data leakage, and data leakage can potentially nullify later analyses that rely on these steps. Can the authors include in the manuscript how they ensured that that no data leakage occurred during their analyses?
   - Answer 7 (to reviewer 2): The sentence is : “The window width of 1.5 mm was selected as a compromise between noise reduction and spatial resolution, on healthy corneas where steps A–G steps was performed automatically without need for manual correction, we observed that the depth-intensity profiles extracted was constant across the 6 mm region of interest for this window width.”
   - We believe the confusion comes from “The window width of 1.5 mm was selected […] on healthy corneas”, we didn’t mean to insinuate there was a difference on the processing, and there is in fact no difference in this process, all images a processed similarly. The sentence here missed a “break” : The window width of 1.5 mm was selected as a compromise […]**. O**n healthy corneas”,
   - We can reformulate for the sake of clarity: “The window width of 1.5 mm was selected as a compromise between noise reduction and spatial resolution. This parameter **was determined a priori** and applied uniformly to all samples. **In healthy corneas, where steps A–G were performed fully automatically without manual correction, the resulting depth–intensity profiles were observed to be consistent across the 6 mm region of interest for this window width.”**
   - Image processing and feature extraction were conducted after preprocessing using the same procedure for all samples.
8. Comment 8 reviewer 2: Line 301: symbol *alpha*
   - Answer 8 (to reviewer 2): corrected thank you
9. Comment 9 reviewer 2: Line 340: Sentence structure:
   - Answer 9 (to reviewer 2): changing sentence “First images are acquired by professionals trained in OCT acquisition and are match with the clinical quality standards” to “images are acquired by professionals trained in OCT acquisition and meet established clinical quality standards”
10. Comment 10 reviewer 2: Line 382: Can the authors provide data on how many patients were the images collected from, and how many images belonged to the same patient?
    - Answer 10 (to reviewer 2): Thank you for this comment; table 1 edited, we also added a column for the acquisition mode and a detail on pixel size, as it was also asked in another comment.
11. Comment 11 reviewer 2: Line 434: Can the authors clarify whether “unknown” images are a different image dataset than the “FECD” images described previously?
    - Answer 11 In this approach, during training there is no other image than healthy images: unknown images mean image the have never be seen including potential FECD images. Considering next reviewer comment we agreed on removing this part.
12. Comment 12 reviewer 2: Line 433-438: While using SVM to create a reference distribution using single label data can work in some scenarios, it’s not a widely used method, compared to let’s say, Gaussian Discriminative Analysis. As such, this section does not seem to contribute critical information and I would suggest removing it and only focusing on the two-label training.
    - Answer 12 (to reviewer 2): We agreed on removing this part.
13. Comment 13 reviewer 2: Line 452: *training*:
    - Answer 13 (to reviewer 2): corrected thank you
14. Comment 14 reviewer 2: Line 460: Accuracy is not a valid metric for imbalanced datasets, so I would suggest removing it.
    - Answer 14 (to reviewer 2): we agreed
15. Comment 15 reviewer 2: Line 460: Which RF model out of the 5 cross-validated models was used to evaluate the test set?
    - Answer 15 (to reviewer 2): Cross-validation was used on the training set to compare candidate models and estimate their performance. The selected model (Random Forest) was then re-instantiated and retrained on the full training dataset using the chosen hyperparameters. This final model was subsequently evaluated on the independent test set.
    - We propose the clarification: “[…] Python [25]. **Model selection was performed using 5-fold stratified cross-validation on the training set, based on the weighted F1-score, to compare candidate classifiers. The random forest model consistently achieved the best performance and was therefore selected. Following this selection, the final model was re-instantiated and retrained on the full training dataset using the chosen hyperparameters**. On the held-out test set […]”
16. Comment 16 reviewer 2: Line 462: I am concerned about the extremely high ROC and predictive power of the model. The results are “too good to be true” and indicate that this is essentially a trivial problem. Since the authors have previously stated that it is not easy to use the images to detect FECD and haze, while at the same time, a random forest model can achieve an ROC of 99.8%, there appears to be a mismatch. I would encourage the authors to carefully review their data generation process to identify and eliminate any “hidden” sources of data leakage.
    - Answer 16 (to reviewer 2): We understand the reviewer's comment, and carefully investigated for an eventual leakage, however images are processed similarly for each condition, devices, and practitioners are shared for each condition. We also want to highlight that we trained and tested the model on a set of images and further tested it as presented in the manuscript on repeatability analysis for both condition-varying practitioners. In our introduction, we emphasized the need for quantitative, objective, and reproducible, not hard we focus firstly on the need for objective, quantitative and reproducible analysis. It is now accepted that machine learning can accurately reproduce human judgement in many cases when done carefully, as we tried to do here. This is still a proof of principle, however, and evaluation on larger, multicentric datasets acquired by diverse acquisition devices, protocols, and practitioners may raise additional challenges
17. Comment 17 reviewer 2: Fig 3a: I have not previously come across this plot format for classification results, and therefore am not able to understand it. It will be helpful to explain the plot in more detail for interpretability.
    - Answer 17 (to reviewer 2): the Y-axis often represents kernel density estimate (KDE) width, sometimes called “density” or “Probability density”. We propose to edit panel a legend from : “distribution of predicted fibrosis probabilities ($p_{\mathrm{FECD}}$) (Healthy mean predicted probability 10.0\% std: 13.2\%; Fuchs' Dystrophy mean predicted probability: 90.9\% std: 14.2\%; Haze mean predicted probability 96.0\% std: 4.7\%).” To “Distribution of predicted FECD probabilities ($p_{\mathrm{FECD}}$) for healthy corneas, FECD, and visual-haze images in the independent test set. The horizontal axis corresponds to the model-estimated probability of FECD represented by the violin curve. Healthy samples cluster near 0, whereas FECD and visual-haze samples cluster near 1 (healthy mean predicted probability 10.0\% std: 13.2\%; Fuchs' Dystrophy mean predicted probability: 90.9\% std: 14.2\%; Haze mean predicted probability 96.0\% std: 4.7\%).”
    - Additionally in the text we edited “reveals a clear separation between the control and pathological groups” to “reveals a clear separation in the x-axis (FECD probability) between the control and pathological groups (y-axis being the model-estimated probability of FECD represented by the violin curve for data distribution visualization).”
    - We renamed the y-axis to “Kernel density estimate (a.u.)” for clarity
18. Comment 18 reviewer 2: Paragraph 500: It’s unclear how the probability associates with real patient outcomes in the longitudinal study. There does not appear to be any medical diagnosis to establish “ground truth”, which can be compared with the model predictions.
    - Answer 18 (to reviewer 2): This is an exploratory analysis, investigating further uses of the proposed model. It will need to be strengthened by a proper longitudinal study where a clinical baseline would be constructed for a proper clinical evaluation. As we are exploring the potential application of the automatic and reproducible quantification we are proposing.
19. Comment 19 reviewer 2: Paragraph 524: Since a primary contribution of the manuscript is “interpretability” of the models, I would suggest improving this section by expanding how the Shap values relate to the attributes of the disease and whether the high-importance features align with the expectations of the medical professionals.
    - Answer 19 (to reviewer 2): The nine quantifiers have been build to probe the specific features described the 3 morphological feature described in “Morphological parameters of subepithelial fibrosis”. We propose to edit the l528 : « higher values of peak width, sigma, area ratio, and depth; and lower values of beta (reduced stromal intensity signal decay) are associated with higher SHAP values” by adding: “Those parameters relating by construction to the 3 morphological parameters of subepithelial fibrosis : sub-epithelial hyper reflectivity (link to area ratio), blurred epithelium/stroma interface (link to peak width, sigma and depth), altered reflectivity depth profile, stromal transparency (link to beta).”
20. Comment 20 reviewer 2: All figures: Can the authors ensure that the figures in the final publication are high quality as the ones in the manuscript are very low-resolution and difficult to read, though it may be an artifact of journal post-processing?
    - Answer 20 (to reviewer 2): Indeed, the pixelated appearance is an artefact of the journal’s post-processing: following the editorial policy, images are removed from the manuscript file and uploaded individually as high-resolution TIFF files (>600 dpi), which the editorial system inserts automatically at full resolution in the compiled PDF. In this resubmission, per the editorial office’s request, the figures have been removed from within the manuscript file (captions retained) and are provided as separate TIFF image files, ensuring that the versions appearing in the final publication are of full quality.
