# Supplementary material for: Machine learning quantitative OCT derived fibrosis indicators for corneal haze evaluation
Source: PLoS One. 2026 Oct 22;21(10):e0328320. doi: 10.1371/journal.pone.0328320 (PMC13690194; doi:10.1371/journal.pone.0328320)
Supplement: Peer review history

# Peer review: Decision Letter 0

**DOI:** 10.1371/journal.pone.0328320.r001

**Authors:** Georgios Labiris

28 Dec 2025

PONE-D-25-32169

Explainable machine learning for post PKR surgery follow-up

PLOS One

Dear Dr. Soubeiran,

Thank you for submitting your manuscript to PLOS ONE. After careful consideration, we feel that it has merit but does not fully meet PLOS ONE’s publication criteria as it currently stands. Therefore, we invite you to submit a revised version of the manuscript that addresses the points raised during the review process.

Both reviewers identify substantial concerns regarding clarity, methodological transparency, and reproducibility that must be addressed before the manuscript can be considered for publication. For acceptance, the authors are required to clearly and consistently define the study objective across the title, abstract, and main text; explicitly describe the datasets, labeling strategy, and training/validation/testing design; and provide sufficient methodological detail (including image processing steps, feature definitions, mathematical formulations, and machine learning procedures) to allow independent replication, in line with PLOS ONE’s criteria for methodological rigor and transparency. Figures and tables that are currently unclear or incomplete must be revised or removed, and terminology should be made consistent throughout. Recommended revisions include expanding the clinical and technical background in the Introduction to improve accessibility for a broad readership and strengthening the literature context. Where reviewer comments overlap (e.g., on unclear methodology), these should be addressed comprehensively; where they diverge, priority should be given to ensuring reproducibility and clarity rather than perceived novelty or impact. The decision for Major Revision is based solely on the need to meet PLOS ONE’s standards for clarity, rigor, and reproducibility.

Please submit your revised manuscript by Feb 11 2026 11:59PM. If you will need more time than this to complete your revisions, please reply to this message or contact the journal office at plosone@plos.org. When you're ready to submit your revision, log on to https://www.editorialmanager.com/pone/ and select the 'Submissions Needing Revision' folder to locate your manuscript file.

Please include the following items when submitting your revised manuscript:

- A letter that responds to each point raised by the academic editor and reviewer(s). You should upload this letter as a separate file labeled 'Response to Reviewers'.
- A marked-up copy of your manuscript that highlights changes made to the original version. You should upload this as a separate file labeled 'Revised Manuscript with Track Changes'.
- An unmarked version of your revised paper without tracked changes. You should upload this as a separate file labeled 'Manuscript'.

If you would like to make changes to your financial disclosure, please include your updated statement in your cover letter. Guidelines for resubmitting your figure files are available below the reviewer comments at the end of this letter.

If applicable, we recommend that you deposit your laboratory protocols in protocols.io to enhance the reproducibility of your results. Protocols.io assigns your protocol its own identifier (DOI) so that it can be cited independently in the future. For instructions see: https://journals.plos.org/plosone/s/submission-guidelines#loc-laboratory-protocols. Additionally, PLOS ONE offers an option for publishing peer-reviewed Lab Protocol articles, which describe protocols hosted on protocols.io. Read more information on sharing protocols at https://plos.org/protocols?utm_medium=editorial-email&utm_source=authorletters&utm_campaign=protocols.

We look forward to receiving your revised manuscript.

Kind regards,

Georgios Labiris, MD, PhD

Academic Editor

PLOS One

Journal Requirements:

When submitting your revision, we need you to address these additional requirements.

1. Please ensure that your manuscript meets PLOS ONE's style requirements, including those for file naming. The PLOS ONE style templates can be found at

https://journals.plos.org/plosone/s/file?id=wjVg/PLOSOne_formatting_sample_main_body.pdf and

https://journals.plos.org/plosone/s/file?id=ba62/PLOSOne_formatting_sample_title_authors_affiliations.pdf

2. Please note that PLOS One has specific guidelines on code sharing for submissions in which author-generated code underpins the findings in the manuscript. In these cases, we expect all author-generated code to be made available without restrictions upon publication of the work. Please review our guidelines at https://journals.plos.org/plosone/s/materials-and-software-sharing#loc-sharing-code and ensure that your code is shared in a way that follows best practice and facilitates reproducibility and reuse.

3. Thank you for stating the following in the Acknowledgments Section of your manuscript:

“This study was partially funded through the “Prix de la Banque Francaise des Yeux 2022" obtained by Anatole Chessel.”

We note that you have provided funding information that is not currently declared in your Funding Statement. However, funding information should not appear in the Acknowledgments section or other areas of your manuscript. We will only publish funding information present in the Funding Statement section of the online submission form.

Please remove any funding-related text from the manuscript and let us know how you would like to update your Funding Statement. Currently, your Funding Statement reads as follows:

“This study was partially funded through the “Prix de la Banque Francaise des Yeux 2022" obtained by Anatole Chessel.”

Please include your amended statements within your cover letter; we will change the online submission form on your behalf.

4. We note that the grant information you provided in the ‘Funding Information’ and ‘Financial Disclosure’ sections do not match.

When you resubmit, please ensure that you provide the correct grant numbers for the awards you received for your study in the ‘Funding Information’ section.

5. Thank you for stating in your Funding Statement:

“This study was partially funded through the “Prix de la Banque Francaise des Yeux 2022" obtained by Anatole Chessel.”

Please provide an amended statement that declares *all* the funding or sources of support (whether external or internal to your organization) received during this study, as detailed online in our guide for authors at

http://journals.plos.org/plosone/s/submit-now.  Please also include the statement “There was no additional external funding received for this study.” in your updated Funding Statement.

Please include your amended Funding Statement within your cover letter. We will change the online submission form on your behalf.

6. Thank you for stating the following financial disclosure:

“This study was partially funded through the “Prix de la Banque Francaise des Yeux 2022" obtained by Anatole Chessel.”

Please state what role the funders took in the study.  If the funders had no role, please state: "The funders had no role in study design, data collection and analysis, decision to publish, or preparation of the manuscript."

If this statement is not correct you must amend it as needed.

Please include this amended Role of Funder statement in your cover letter; we will change the online submission form on your behalf.

7. We note that you have indicated that there are restrictions to data sharing for this study. PLOS only allows data to be available upon request if there are legal or ethical restrictions on sharing data publicly. For more information on unacceptable data access restrictions, please see http://journals.plos.org/plosone/s/data-availability#loc-unacceptable-data-access-restrictions.

Before we proceed with your manuscript, please address the following prompts:

a) If there are ethical or legal restrictions on sharing a de-identified data set, please explain them in detail (e.g., data contain potentially identifying or sensitive patient information, data are owned by a third-party organization, etc.) and who has imposed them (e.g., a Research Ethics Committee or Institutional Review Board, etc.). Please also provide contact information for a data access committee, ethics committee, or other institutional body to which data requests may be sent.

b) If there are no restrictions, please upload the minimal anonymized data set necessary to replicate your study findings to a stable, public repository and provide us with the relevant URLs, DOIs, or accession numbers. For a list of recommended repositories, please see

https://journals.plos.org/plosone/s/recommended-repositories. You also have the option of uploading the data as Supporting Information files, but we would recommend depositing data directly to a data repository if possible.

We will update your Data Availability statement on your behalf to reflect the information you provide.

8. Your ethics statement should only appear in the Methods section of your manuscript. If your ethics statement is written in any section besides the Methods, please move it to the Methods section and delete it from any other section. Please ensure that your ethics statement is included in your manuscript, as the ethics statement entered into the online submission form will not be published alongside your manuscript.

9. Please remove your figures from within your manuscript file, leaving only the individual TIFF/EPS image files, uploaded separately. These will be automatically included in the reviewers’ PDF.

10. If the reviewer comments include a recommendation to cite specific previously published works, please review and evaluate these publications to determine whether they are relevant and should be cited. There is no requirement to cite these works unless the editor has indicated otherwise.

[Note: HTML markup is below. Please do not edit.]

Reviewers' comments:

Reviewer's Responses to Questions

Comments to the Author

1. Is the manuscript technically sound, and do the data support the conclusions?

The manuscript must describe a technically sound piece of scientific research with data that supports the conclusions. Experiments must have been conducted rigorously, with appropriate controls, replication, and sample sizes. The conclusions must be drawn appropriately based on the data presented.

Reviewer #1: Partly

Reviewer #2: Partly

2. Has the statistical analysis been performed appropriately and rigorously?

Reviewer #1: Yes

Reviewer #2: I Don't Know

3. Have the authors made all data underlying the findings in their manuscript fully available?

The PLOS Data policy requires authors to make all data underlying the findings described in their manuscript fully available without restriction, with rare exception (please refer to the Data Availability Statement in the manuscript PDF file). The data should be provided as part of the manuscript or its supporting information, or deposited to a public repository. For example, in addition to summary statistics, the data points behind means, medians and variance measures should be available. If there are restrictions on publicly sharing data—e.g. participant privacy or use of data from a third party—those must be specified.

Reviewer #1: No

Reviewer #2: No

4. Is the manuscript presented in an intelligible fashion and written in standard English?

PLOS ONE does not copyedit accepted manuscripts, so the language in submitted articles must be clear, correct, and unambiguous. Any typographical or grammatical errors should be corrected at revision, so please note any specific errors here.

Reviewer #1: No

Reviewer #2: Yes

5. Review Comments to the Author

Please use the space provided to explain your answers to the questions above. You may also include additional comments for the author, including concerns about dual publication, research ethics, or publication ethics. (Please upload your review as an attachment if it exceeds 20,000 characters)

Reviewer #1: This manuscript deals with an important clinical issue. It proposes hand-crafted features extracted from OCT images and uses classical deep learning techniques to identify clinically abnormal corneal conditions. However, serious issues exist, which need to be addressed before reconsidering for publication.

One major issue of this manuscript is the lack of clarity about the aim and the objective. The title is very unclear as “machine learning for PRK follow up”.

In Line 149 - 150 it is stated “Our final goal is an automatic classification of clinical OCT images into two categories: "healthy corneas" and "corneal haze"”. This statement, if it holds, it should be made very early in the manuscript.

In the abstract it is stated “we trained the model on corneas presenting Fuchs’ dystrophy, causing similar, but permanent, fibrosis symptoms, and applied it to images from patients who have undergone PRK surgery”. Immediately after that is follows “… the model output (probability of Fuchs’ dystrophy classification) …”

Then, results are presented using 5-cross validation on the FECD images. In Table 1 several databases are mentioned without explaining the training set and the test set from which results are presented.

Moreover, in Fig. 5 repeatability results are presented from 10 healthy and 5 Fuchs retinas, without explaining if these are different from the 166 healthy, 313 Fuch retinas, or the remaining Haze retinas of Table 5.

Thus it is unclear to the reader what is the aim of the work.

The authors should modify the title and rewrite the abstract to make it more specific. Following the introduction, a short subsection entitled “Objective and contributions” (or similar) should be added describing exactly that, without any ambiguity. Table 2 should be redesigned to clarify all the above.

Fig. 4a is completely unclear. The “distribution” axis appears to have no meaning. The blue and red areas are not explained. Please either describe the figure extensively in the text, explaining every aspect, or remove it from the manuscript, since in its current form it only creates questions to the readers.

Subsection 3.2, Lines 84 - 95 “the processing chain described in Ref. 2. This pipeline is supplemented with an additional alignment procedure relying on normalised cross-correlation”

The authors must include the exact steps that are followed, both for [2], as well as for the alignment procedure and the rest of the steps, since the above are non-trivial image processing tasks. If this is too technical for the journal, then some form of additional material should be used. In its current form, the statement is not acceptable for publication, since the methodology is obscure and researchers cannot dublicate the procedures.

Caption of Fig. 2: “red square” --> “red rectangle”

Subsection 3.3

The descriptions are very brief, without definitions and clear steps. Just to mention some confusing points:

There is no clear description how the image in Fig. 3b is generated from Fig.3a

The “depth-averaged profile” is calculated along the Y-axis, or along the normal vector to the corneal surface ? Please define explicitly.

Every step of the process after definition of the profile is based on clearly identifiable landmarks of the profile (first anterior maximum, 2nd maximum, minimum before and after the 2nd maximum, etc). It seems from the manuscript that if these landmarks are not correctly detected, the remaining process will fail. The authors should discuss how will their proposed system compensate for depths with increased SNR, where the profile maybe very noisy preventing the detection of these curve landmarks.

In Line 134 the exponential regressor has a constant term C, which is missing from Fig. 3f.

In Fig. 3e the MSE is wrong (summation over the samples is missing, division be the number of samples).

As a general comment, mathematical quantities should be rigorously defined in the manuscript in numbered Equations, with every symbol explained. Please remove the incomplete, hard-to-read mathematical definitions from the figures.

Line 110: “An interpolation is performed to improve the sampling in this area …”:

Once again, please define clearly every aspect of the step.

Define the calculation of “Area ratio”. The relevant lines 141-143 are completely confusing.

Subsection 4.3

The purpose of the subsection is unclear. Is there any connection with the proposed system ? Please clarify

Fig. 6

Please define the calculation of SNR of the OCT image, the effect it has in the prediction. Also provide the numerical values.

Line 236, 260 “FD class” is the same as “FECD” class ? If yes, please replace.

Line 236 why is “additional training” mentioned ? Has it been used or discussed somewhere else in the manuscript ?

Fig. S3, right column: please explain in the text how are the figures generated, from how many images and how are μ, σ calculated and from which specific images in each figure

Reviewer #2: Soubeiran et al have presented a machine learning based approach to classify healthy corneas vs those with Fuchs’ dystrophy. The study setup seems appropriate and the results are promising.

However, as a general PLOS One reader unfamiliar with this medical field, I was unable to fully follow the manuscript and found the description of both the disease and the methodology lacking for a comprehensive review. I have described both my major and minor concerns below:

1. As PLOS One caters to a diverse audience, it would be helpful if the authors presented more information about the medical condition and the problem motivation. I appreciate the need to keep the manuscript succinct, and do not expect a full background of every medical term. However using simpler language, describing the most relevant terms, and emphasizing the motivation of this study would make the manuscript more broadly accessible.

2. The methodologies are not described sufficiently for a comprehensive review. For example, data cleaning methodologies are only subjectively described. Feature ranges are missing. Image labeling methodology is missing. Hyperparameter selection is missing.

3. Line 15 - Suggest including the name of the machine learning algorithm in the title, abstract, and Introduction. ML is too broad a term to describe the authors’ specific work.

4. Line 28 - It is not clear why the authors have started the Introduction with a reference to their own previous work. While both works relate to Fuchs, the previous work has not included any ML. I would suggest starting the Introduction with the specific problem being solved in this work, and referencing previous work in a later paragraph.

5. Introduction - There is almost no literature review in Introduction. The authors have claimed that they are not aware of quantitative tools but they have not provided sufficient data to support their statement. Additionally, even if quantitative tools are not available, surely there would be other work related to Fuchs that motivates the authors’ work and highlights why haze diagnosis is required. Other work will also serve as a comparison for how the authors’ ML model improves upon existing evaluation performance.

6. Line 49 - What are the missing physical parameters?

7. Fig 1 - What are Ref 3 and Ref 6?

8. Line 56 - Please specify the algorithm instead of broad term like “automatic classification approach”.

9. Line 60 - The sentence is unclear.

10. Line 70 - The sentence is unclear.

11. Line 74 - What segmentation algorithms are being referred to? If there are segmentation algorithms applied here, how does this relate to authors’ pervious statement about missing quantitative tools?

12. Line 75 - What is the issue with “overestimation of the epithelial layer thickness”?

13. Line 78 - Since the “marked morphological features” are an essential input in the authors’ model, it is insufficient to refer to their description in another reference. I would suggest including a description in this manuscript.

14. Line 85 - What is Ref 2?

15. Line 85 - What is the “additional alignment procedure”?

16. Line 87 - How much time and effort is required to detect the “central saturation artefact”? If its detection is essential to leveraging the authors’ methodology, would the image analysis already be done, thus making their approach extraneous?

17. Section 3.2 - Would these image processing capabilities be available to physicians who intend to use the authors’ methodology?

18. Line 90 - Please include more description and reference for PCA.

19. Line 90 - How are low intensity regions defined and removed?

20. Line 91 - How is 1.5 mm chosen?

21. Line 93 - What is the functional form of the weighting function? How is the weight range chosen?

22. Fig 3 - “peak Area Ratio” is not described in Fig 3d.

23. Line 119 - What is being defined in item 4?

24. Line 143 - What is meant by “available simultaneously”?

25. Line 153 - It would be helpful if the authors provided evidence about how the classification model applied to FECD images justifies the model’s application for corneal haze detection.

26. Line 155 - Are the 9 quantifiers the features for the ML models?

27. Line 164 - How would the doctors label the images? Would they use any of the 9 quantifiers to make their determination?

28. Line 169 - “very precisely” is a subjective term, and not descriptive in this context.

29. Line 170 - How did the authors train SVM, determine its hyperparameters, and experiment with reducing overfitting?

30. Line 172 - Does this mean that no label separation was done for implementation of SVM? How can the models performances be compared if they were trained with different datasets?

31. Line 177 - How did the authors select 50% as the probability threshold, especially because the training dataset is unbalanced?

32. Line 184 - After cross-validation, since there will be 5 trained models, which one of the models was applied to the test set?

33. Line 186 - The sentence structure makes it seem that the high TPR and FPR at a single threshold contributes to the high ROC, which is inaccurate since ROC is generated over the entire threshold range.

34. Line 218 - Suggest changing AI to ML to remain consistent with the rest of the manuscript.

6. PLOS authors have the option to publish the peer review history of their article (what does this mean?). If published, this will include your full peer review and any attached files.

If you choose “no”, your identity will remain anonymous but your review may still be made public.

Do you want your identity to be public for this peer review? For information about this choice, including consent withdrawal, please see our Privacy Policy.

Reviewer #1: No

Reviewer #2: No

[NOTE: If reviewer comments were submitted as an attachment file, they will be attached to this email and accessible via the submission site. Please log into your account, locate the manuscript record, and check for the action link "View Attachments". If this link does not appear, there are no attachment files.]

To ensure your figures meet our technical requirements, please review our figure guidelines: https://journals.plos.org/plosone/s/figures

You may also use PLOS’s free figure tool, NAAS, to help you prepare publication quality figures: https://journals.plos.org/plosone/s/figures#loc-tools-for-figure-preparation.

NAAS will assess whether your figures meet our technical requirements by comparing each figure against our figure specifications.

# Author comment: Author response to Decision Letter 1

**DOI:** 10.1371/journal.pone.0328320.r002

5 Mar 2026

We sincerely thank Reviewers for their detailed, constructive comments. We have carefully revised the manuscript in response to all points raised. Below, we provide a point-by-point response. All changes are indicated in the revised manuscript; revised line numbers refer to the resubmitted version. Detailed answers are formatted into the "Response to Reviewer" document.

Reviewer #1:

1. Comment: One major issue of this manuscript is the lack of clarity about the aim and the objective. The title is very unclear as “machine learning for PRK follow up”.

In Line 149 - 150 it is stated “Our final goal is an automatic classification of clinical OCT images into two categories: "healthy corneas" and "corneal haze"”. This statement, if it holds, it should be made very early in the manuscript.

In the abstract it is stated “we trained the model on corneas presenting Fuchs’ dystrophy, causing similar, but permanent, fibrosis symptoms, and applied it to images from patients who have undergone PRK surgery”. Immediately after that is follows “… the model output (probability of Fuchs’ dystrophy classification) …”

Then, results are presented using 5-cross validation on the FECD images. In Table 1 several databases are mentioned without explaining the training set and the test set from which results are presented.

Moreover, in Fig. 5 repeatability results are presented from 10 healthy and 5 Fuchs retinas, without explaining if these are different from the 166 healthy, 313 Fuch retinas, or the remaining Haze retinas of Table 5.

Thus it is unclear to the reader what is the aim of the work.

The authors should modify the title and rewrite the abstract to make it more specific. Following the introduction, a short subsection entitled “Objective and contributions” (or similar) should be added describing exactly that, without any ambiguity. Table 2 should be redesigned to clarify all the above.

a. Answer: We understand that the initial version of the manuscript did not sufficiently clarify the aim, workflow, and dataset usage, which could lead to confusion regarding the objective and evaluation strategy. In the revised manuscript, we clarified the objective: The title has been refined to better reflect the scope of the study: derive fibrosis indicators. We believe we now explicitly state that the primary goal is the automatic classification of corneal OCT images into healthy vs. fibrotic (haze-like) corneas, using physics-based quantifiers and a machine learning model. We explain the rationale of training on FECD images (as a proxy for fibrosis) and applying the model to PRK follow-up data.

b. In details, we added in the abstract a precision about the transient nature of the fibrosis and the link with Fuchs’ Dystrophy (and shared symptoms), and clarified the use of OCT-derived quantifiers. As the reviewer reported confusion and a structural problem in the introduction. We performed a major revision of the introduction. We now start by the source of the corneal Haze the PKR surgery, describing its clinical symptoms and presenting the current means of evaluation (revision l1-24). We explain why OCT may be more reliable for quantitative assessment (revision l26-29). We then describe the transient nature of the fibrosis occurring in Haze and why Fuch’s Dystrophy is interesting, as it shares similar symptoms but is permanent and therefore suitable to create a bigger database to access such symptoms (revision l30-42). We conclude the introduction by a clear statement of our aim (address the lack of objective haze quantification) and hypothesis. To remove ambiguity, we introduced a dedicated paragraph at the end of the Introduction (revision l43–53) explicitly stating the objective and hypothesis of the study. This paragraph clearly distinguishes: the training/validation task (classification of FECD vs. healthy corneas), from the application task (transfer to PRK haze assessment).

c. We also clarified the use of datasets throughout the manuscript. In the revised Materials and Methods and Results sections, we explicitly describe: the composition of each dataset (healthy, FECD, PRK), the role of each dataset (training, validation, independent testing), and the cross-validation strategy (revision l376–383, Table 1 updated accordingly). By defining each cohort successively we explain what they address (revision l372-404) and kept the same order in results (revision l332-516).

d. Regarding the confusion on specific figures: For Fig. 5 (repeatability), we now explicitly state that the subset “10 healthy subjects (10 corneas × 5 repetitions) and 3 patients with Fuchs’ Dystrophy (5 corneas × 5 repetitions)” (revision l472-473), we believe the the new structuration explicit the independent subset used exclusively for repeatability analysis, distinct from the training and validation cohorts. We clarified these distinctions in the figure caption and corresponding text, and Table 1 (and related tables) has been redesigned to clearly indicate dataset splits, sample sizes, and their respective roles (training, validation, testing,), ensuring full transparency and reproducibility.

e. We understand that this comment was broader then the listed points so we added other modifications such as clarifying the processing pipeline (Steps A-H), the quantifiers construction (and their link to clinical insight), precised the processing time (l327-335), robustness (l337-354).

2. Comment: Fig. 4a is completely unclear. The “distribution” axis appears to have no meaning. The blue and red areas are not explained. Please either describe the figure extensively in the text, explaining every aspect, or remove it from the manuscript, since in its current form it only creates questions to the readers.

a. Answer: Thank you for this remark. We renamed the axis (“probability density”), and the colors are detailed in the legend.

3. Comment: Subsection 3.2, Lines 84 - 95 “the processing chain described in Ref. 2. This pipeline is supplemented with an additional alignment procedure relying on normalised cross-correlation”

The authors must include the exact steps that are followed, both for [2], as well as for the alignment procedure and the rest of the steps, since the above are non-trivial image processing tasks. If this is too technical for the journal, then some form of additional material should be used. In its current form, the statement is not acceptable for publication, since the methodology is obscure and researchers cannot dublicate the procedures.

a. Answer: We agree. In the revised version, we have addressed this concern by explicitly detailing the complete processing chain within the manuscript (Section Materials and Methods, revision l112–220). All steps (A to H) are now described, including preprocessing, artefact detection and removal, anterior surface detection, A-scan alignment, stromal segmentation, PCA-based artefact correction, lateral normalization, and depth-intensity profile extraction. In particular, the alignment procedure previously referenced has now been fully described (revision l150). To further improve clarity and reproducibility, we ensured that all algorithmic parameters (e.g., filter sizes, thresholds, window widths) are specified in the text, and we provide mathematical formulations where applicable. As a result, the methodology is now self-contained within the manuscript and does not rely on external references for critical steps.

4. Comment: Caption of Fig. 2: “red square” --> “red rectangle”

a. Answer: Done

5. Comment: Subsection 3.3 The descriptions are very brief, without definitions and clear steps. Just to mention some confusing points:

There is no clear description how the image in Fig. 3b is generated from Fig.3a

The “depth-averaged profile” is calculated along the Y-axis, or along the normal vector to the corneal surface ? Please define explicitly.

Every step of the process after definition of the profile is based on clearly identifiable landmarks of the profile (first anterior maximum, 2nd maximum, minimum before and after the 2nd maximum, etc). It seems from the manuscript that if these landmarks are not correctly detected, the remaining process will fail. The authors should discuss how will their proposed system compensate for depths with increased SNR, where the profile maybe very noisy preventing the detection of these curve landmarks.

a. Answer: Thank you for this comment we therefore reworked this section 3.3, which now corresponds to “Definition of subepithelial fibrosis quantifiers” section l229, additionally to the pipeline details we added in Step A-H. The definitions of quantifiers is now clearer and we mention their meaning. Additionally, regarding the implementation itself, we added details and “Processing time” (l327). We also want to mention that the code will be available upon publication for the reader as required by PlosOne.

b. Step C now explain the figure 2 (replacing figure 3) a to b. We understand that this remark was wider than just this point, and we therefore meticulously detailed the other steps and quantifiers.

c. Regarding landmark detection, we addressed this point in a new paragrapher “Robustness to low-SNR conditions and landmark detection failures” l337

6. Comment: In Line 134 the exponential regressor has a constant term C, which is missing from Fig. 3f.

a. Answer: Added

7. Comment: In Fig. 3e the MSE is wrong (summation over the samples is missing, division be the number of samples).

As a general comment, mathematical quantities should be rigorously defined in the manuscript in numbered Equations, with every symbol explained. Please remove the incomplete, hard-to-read mathematical definitions from the figures.

a. Answer: Thank you, the MSE was displaying the upper bar that is the mathematical symbol of the mean that includes the mentioned division. However, we agree that more mathematical formalism would clarify the manuscript, we added all the formulas, including the MSE (equation 2 l279-280) that clarify the bar notation and explexitely mention it at l282. We understand the fact that MSE and COV were hard to read in figure 2 (replacing fig3), but as we want this figure to display the 9 quantifiers, we keep it by putting it in a box and coloring the 9 quantifiers in red.

8. Comment: Line 110: “An interpolation is performed to improve the sampling in this area …”:

Once again, please define clearly every aspect of the step.

Define the calculation of “Area ratio”. The relevant lines 141-143 are completely confusing.

a. Answer: We now describe better in Step H (l202-228) the average profile extraction. The area ratio is also now explicated in the figure 2 (replacing fig 3) and in the text (l309)

9. Comment: Subsection 4.3 The purpose of the subsection is unclear. Is there any connection with the proposed system ? Please clarify

a. Answer: As now we describe the cohort and their purpose from the method section we believe this aspect has been clarified (l398-404 and l512-590 for the results). We clarified for instance with l401:“This to study the transient evolution of post surgical inflammation and fibrosis, and to evaluate how our quantifiers track the progression and resolution of corneal haze and fibrosis indicators over time, providing insights into the healing process and the potential for our quantifiers to monitor disease progression and resolution in a clinical setting.“, and l521“It suggests patient-specific trajectories of recovery that could be further investigated in larger cohorts to identify potential prognostic factors and inform personalized management strategies for patients undergoing PRK surgery.“. We believe these results have a place here since Haze is transient.

10. Comment: Fig. 6 Please define the calculation of SNR of the OCT image, the effect it has in the prediction. Also provide the numerical values.

a. Answer: Thank you, equation 6 has been added and described l483-487, legend edited snr values scale on each figure have the same scale, but we agree it needs to be in the legend for each one.

11. Comment: Line 236, 260 “FD class” is the same as “FECD” class ? If yes, please replace.

Line 236 why is “additional training” mentioned ? Has it been used or discussed somewhere else in the manuscript ?

a. Answer: Thank you, FD has been edited to FECD in the revision

b. This meant that once the model was trained on healthy/FECD corneas we presented it with Haze cornea (and not re-train it on those images) it was a methodological point. Has this was unclear, we detailed each cohort more clearly in the material and methods section, and present in the same order in the results section. For this specific point this result in exposed now in revision l488-499, and the corresponding sentence edited to “The trained model was applied to the 6 images from patients that underwent PRK surgery and reporting vizual haze (application cohort from Table 1).” At l489

12. Comment: Fig. S3, right column: please explain in the text how are the figures generated, from how many images and how are μ, σ calculated and from which specific images in each figure

a. Answer: We included this remark in the revision by defining those parameters and detailing the image generation.

Reviewer 2:

1. Comment: As PLOS One caters to a diverse audience, it would be helpful if the authors presented more information about the medical condition and the problem motivation. I appreciate the need to keep the manuscript succinct, and do not expect a full background of every medical term. However using simpler language, describing the most relevant terms, and emphasizing the motivation of this study would make the manuscript more broadly accessible.

a. Answer: We reshaped the introduction to introduce more medical background (revision l1-24), we refined the relevance of OCT (revisionl26-42) and clarified the aim (revision l43-53) to make the revised manuscript more accessible in accordance with this comment.

2. Comment: The methodologies are not described sufficiently for a comprehensive review. For example, data cleaning methodologies are only subjectively described. Feature ranges are missing. Image labeling methodology is missing. Hyperparameter selection is missing.

a. Answer: Thank you for those remarks. Overall we tried to clarify in the revision each step of the process to answer this concern. Specifically to those mentioned points: we now give a description of the methodology for the pipeline (Steps A to H, revision l118-220) we specified range values of algorithm varible; the clinical parameters used for labelling are now described (revision l76-90); hyperparameter selection precised in revision l455-459; we detail the clinical meaning of our quantifiers, their equation, range of the quantifiers are available in S1 fig; in to answer this concern.

b. We also understand that this point was more global than just mentioned point; this is we rewrote different sections. The introduction is to make our objective clearer and give more context on the clinical assessment, the automatic approach of the literature, and the missing parts. We reworked the material and methods section to clarify the methodology and the datasets. Specifically, we also detailed more extensively each cohort so the results section is more reproducible.

3. Comment: Line 15 - Suggest including the name of the machine learning algorithm in the title, abstract, and Introduction. ML is too broad a term to describe the authors’ specific work.

a. Answer: We refined the title while keeping “machine learning”, although machine learning is indeed a broad term, it still refers to a specific type of algorithm that we specifically tested in this paper and referenced both in the manuscript text (revision l448-450) and in S1 Table. However, to add some nuance on the machine learning vs. deep learning in revision l70-71.

4. Comment: Line 28 - It is not clear why the authors have started the Introduction with a reference to their own previous work. While both works relate to Fuchs, the previous work has not included any ML. I would suggest starting the Introduction with the specific problem being solved in this work, and referencing previous work in a later paragraph.

a. Answer: We agree with this remarker and changed the introduction to start by the source of the corneal Haze the PKR surgery, described it’s clinical symptoms and presented the current means of evaluation. We explain why OCT may be more reliable for quantitative assessment. We then describe the transient nature of the fibrosis occurring in Haze and why Fuch’s Dystrophy is interesting as it shares similar symptoms but permanently and is therefore suitable to create a bigger database to access such symptoms. We conclude the introduction by a clear statement of our aim (address the lack of objective haze quantification) and hypothesis.

5. Comment: Introduction - There is almost no literature review in Introduction. The authors have claimed that they are not aware of quantitative tools but they have not provided sufficient data to support their statement. Additionally, even if quantitative tools are not available, surely there would be other work related to Fuchs that motivates the authors’ work and highlights why haze diagnosis is required. Other work will also serve as a comparison for how the authors’ ML model improves upon existing evaluation performance.

a. Answer: Similarly, we addressed this remark by refactoring the introduction, adding a deeper literature review and clinical context.

6. Comment: Line 49 - What are the missing physical parameters?

a. Answer: The idea was to mention that current means of evaluation are not based on any physical parameters and are referred as “presence of haze”. We can’t list what is missing but can assess that there are none. However, we re-worked this sentence by extending what the ophthalmologist is looking for (revision l14-19) and explained the difference between Slit-lamp (subjective that can’t provide “values” or physical parameter) and OCT (that provide a signal linked to the cornea absorption) (revision l19-21). Finally, we conclude on why there is a need for quantitative/objective assessment (revision l24-25).

7. Comment: Fig 1 - What are Ref 3 and Ref 6?

a. Answer: References are now fixed in the revision (Ref3 = revision file ref [1], Ref 6 = revision file ref [2])

8. Comment: Line 56 - Please specify the algorithm instead of broad term like “automatic classification approach”.

a. Answer: We added a mention to Random Forest in revision l64 corresponding to this sentence in the initial submission.

9. Comment: Line 60 - The sentence is unclear.

a. Answer: We tried to clarify this in revision l70-75 corresponding to this sentence in the initial submission by detailing the difference between deeplearning extracting image features that can’t be interpreted by “human” and features created by humans (interpretable) passed to machine learning for classification.

10. Comment: Line 70 - The sentence is unclear.

a. Answer: We added a better description of the “pipeline” in the revision and deeply explained what we are performing in Step B (revision l127-134) to make this clearer.

11. Comment: Line 74 - What segmentation algorithms are being referred to? If there are segmentation algorithms applied here, how does this relate to authors’ pervious statement about missing quantitative tools?

a. Answer: As a big part of this paragraph was unclear for the reviewer, we re-worked it. For this specific point, we now detail in revision l92-95 that the commercial OCT device has segmentation tools that we detailed. But we mentioned their limitations and are not using them. We think that this new paragrapher from l91to 110 gives a better overview.

12. Comment: Line 75 - What is the issue with “overestimation of the epithelial layer thickness”?

a. Answer: Similarly to the previous answer, we are now detailing l94 this point.

13. Comment: Line 78 - Since the “marked morphological features” are an essential input in the authors’ model, it is insufficient to refer to their description in another reference. I would suggest including a description in this manuscript.

a. Answer: We now include this description in step B detailed in revision l127-137.

14. Comment: Line 85 - What is Ref 2?

a. Answer: References are now fixed in the revision (Ref 2 = [13] in revision)

15. Comment: Line 85 - What is the “additional alignment procedure”?

a. Answer: Now described in Step C, specifically in revision l148 to 155.

16. Comment: Line 87 - How much time and effort is required to detect the “central saturation artefact”? If its detection is essential to leveraging the authors’ methodology, would the image analysis already be done, thus making their approach extraneous?

a. Answer: Now detailed in Step B (l128-136), concerning the time and effort we added a “Processing time” section in revision l327-334.

17. Comment: Section 3.2 - Would these image processing capabilities be available to physicians who intend to use the authors’ methodology?

a. Answer: Code is available at publication but we now provide a detailed pipeline (Step A-H) and processing time information specifically l333.

18. Comment: Line 90 - Please include more description and reference for PCA.

a. Answer: Yes, now in E-F l164-195.

19. Comment: Line 90 - How are low intensity regions defined and removed?

a. Answer: Now in Step D with a precision in footnote p5.

20. Comment: Line 91 - How is 1.5 mm chosen?

a. Answer: Now detailed in revision l203 and 207: “The window width of 1.5mm was selected as a compromise between noise reduction and spatial resolution, on healthy corneas where steps A-G steps was performed automatically without need for manual correction, we observed that the depth-intensity profiles extracted was constant across the 6~mm region of interest for this window width.”

21. Comment: Line 93 - What is the functional form of the weighting function? How is the weight range chosen?

a. Answer: Now detailed in revision l217-219:”This weighting reduces the influence of peripheral windows, where curvature-induced SNR degradation and residual artefacts from the flattening procedure are most pronounced, while still retaining their contribution so as not to discard clinically relevant information”. We also precised the weight formula in equation 1). We also mention in l224-228: “In practice, it would be laborious to evaluate the landmark position across the entire 6mm region of interest; we capitalized on the use off the weighted mean of quantifiers and the extraction of 9 different quantifiers to mitigate the impact of potential landmark detection failures in some windows.”

22. Comment: Fig 3 - “peak Area Ratio” is not described in Fig 3d.

a. Answer: We now include it and named it R.

23. Comment: Line 119 - What is being defined in item 4?

a. Answer: We now start each physical parameter by its name

24. Comment: Line 143 - What is meant by “available simultaneously”?

a. Answer: We meant that once the profile is extracted, we can extract those 9 quantifiers. But we agree it was confusing, we removed this sentence, transitioning with “Once these quantifiers are extracted for each image” in revision l316.

25. Comment: Line 153 - It would be helpful if the authors provided evidence about how the classification model applied to FECD images justifies the model’s application for corneal haze detection.

a. Answer: Thank you for this remark. We now give a clear clinical point in revision l80 to 90 to detail what changes in case of fibrosis before detailing the quantifier and detailing for each one what they are “designed” to detect/measure in this context. Then, when presenting the FECD you are mentioning in this question, we detailed: “due to the transient nature of haze and the difficulty of clinical diagnosis in mild cases, insufficient validated clinical images exist to constitute a robust training database. We therefore adopted a transfer learning strategy: we trained and validated our classification model on FECD as in its advanced stages, presents similar subepithelial fibrosis localized in the anterior stroma.” (revision l358-362). Note also that now in the introduction (revision l35-38) we are citing 3 papers on that topic: “presenting with similar symptoms of blurred vision and glare [9]. The commonality of these structural alterations, particularly the anterior stromal fibrosis, suggests that a quantitative approach developed for one condition might be applicable to the other [8, 10, 11]”

26. Comment: Line 155 - Are the 9 quantifiers the features for the ML models?

a. Answer: Yes we now explicitly mention it from the beginning l64 “classification approach based on a random forest classifier applied to physics-based descriptors of fibrosis extracted from OCT images, as described in the following section.“ and after detailing the quantifiers at l316 “Once these quantifiers are extracted for each image, they are used as input features for the classification model described in the next section”

27. Comment: Line 164 - How would the doctors label the images? Would they use any of the 9 quantifiers to make their determination?

a. Answer: We now mention this explicitly in revision l80-90 by explaining what the doctor use to characterize those images. Then when detailing the quantifier, we explain their link to clinical observation (why we choose/buid them) for instance l273 “his ratio directly quantifies the hyperreflectivity observed clinically in fibrotic corneas and correlates with the density of light-scattering structures, primarily activated myofibroblasts and abnormal collagen within the anterior stroma [2]”

28. Comment: Line 169 - “very precisely” is a subjective term, and not descriptive in this context.

a. Answer: We agree, removed

29. Comment: Line 170 - How did the authors train SVM, determine its hyperparameters, and experiment with reducing overfitting?

a. Answer: We now describe Hyperparametrization in l455-459.

30. Comment: Line 172 - Does this mean that no label separation was done for implementation of SVM? How can the models performances be compared if they were trained with different datasets?

a. Answer: Maybe it was unclear but a classic methodology in the literature if sometime to train SVM in a single class (as the cited reference [20]) (for instance healthy) and then present a new dataset not from the original distribution (for instance pathological corneas FECD or Haze) to detect if it fall “within” or “without” the training distribution. In our case, it did not work, and we mention it here. Has it seemed unclear, we reworked this sentence at l433: “in which only the healthy cornea class is modelled in detail and unknown images are classified according to their deviation from this reference distribution.”

31. Comment: Line 177 - How did the authors select 50% as the probability threshold, especially because the training dataset is unbalanced?

a. Answer: We understand that the mention of the 50% was unclear, additionally in fact this information is not used in the rest of the paper as it was an arbitrary value, we removed this mention as it does not affect any result nor conclusion. We consider this probability as a continuous value that “quantifies the degree of confidence in the classification” (revision l444)

32. Comment: Line 184 - After cross-validation, since there will be 5 trained models, which one of the models was applied to the test set?

a. Answer: We now explicitly mention this in l460 “On the held-out test set, the random forest classifier attained an accuracy “. Additionally, in Table 1 we noted the use of each set, and detailed the exact methodology in revision l376-383.

33. Comment: Line 186 - The sentence structure makes it seem that the high TPR and FPR at a single threshold contributes to the high ROC, which is inaccurate since ROC is generated over the entire threshold range.

a. Answer: We agree it is indeed the reverse ROC taken at one value (threshold) that gives the sensitivity/specificity. We edited the sentence accordingly, removing the causality now in revision l460-463.

34. Comment: Line 218 - Suggest changing AI to ML to remain consistent with the rest of the manuscript.

a. Answer: We strongly agree to replace this part. We also clarified why we specifically use ML in revision l64-75.

Attachment Submitted filename: Response to Reviewers.docx

# Peer review: Decision Letter 1

**DOI:** 10.1371/journal.pone.0328320.r003

**Authors:** Georgios Labiris

12 Apr 2026

Dear Dr. Soubeiran,

The revised manuscript shows clear improvement in clarity and structure; however, both reviewers still identify important issues that must be addressed before acceptance. In particular, the authors should (i) substantially improve figure quality and ensure all figures are publication-ready and correctly integrated into the main text, (ii) provide detailed clarification of the methodological pipeline with specific attention to potential data leakage, cohort structure, and preprocessing steps, and (iii) justify or revise the analytical choices that may not be standard or fully supported (e.g., PCA variance assumptions, SVM-based reference distribution, and accuracy reporting in imbalanced settings). Reviewer 2’s concerns regarding possible data leakage and the unexpectedly high model performance are particularly critical and must be explicitly addressed with additional methodological detail or reanalysis where necessary. Please provide a clearer explanation and corrections to the confusing figure elements (especially Fig. 3a), and improve the interpretability of the results. Minor linguistic and formatting issues can be corrected during revision, but do not affect the decision.

plosone@plos.org. When you're ready to submit your revision, log on to https://www.editorialmanager.com/pone/ and select the 'Submissions Needing Revision' folder to locate your manuscript file.

- A letter that responds to each point raised by the academic editor and reviewer(s). You should upload this letter as a separate file labeled 'Response to Reviewers'.
- A marked-up copy of your manuscript that highlights changes made to the original version. You should upload this as a separate file labeled 'Revised Manuscript with Track Changes'.
- An unmarked version of your revised paper without tracked changes. You should upload this as a separate file labeled 'Manuscript'.

If you would like to make changes to your financial disclosure, please include your updated statement in your cover letter. Guidelines for resubmitting your figure files are available below the reviewer comments at the end of this letter.

If applicable, we recommend that you deposit your laboratory protocols in protocols.io to enhance the reproducibility of your results. Protocols.io assigns your protocol its own identifier (DOI) so that it can be cited independently in the future. For instructions see: https://journals.plos.org/plosone/s/submission-guidelines#loc-laboratory-protocols. Additionally, PLOS ONE offers an option for publishing peer-reviewed Lab Protocol articles, which describe protocols hosted on protocols.io. Read more information on sharing protocols at https://plos.org/protocols?utm_medium=editorial-email&utm_source=authorletters&utm_campaign=protocols.

As the corresponding author, your ORCID iD is verified in the submission system and will appear in the published article. PLOS supports the use of ORCID, and we encourage all coauthors to register for an ORCID iD and use it as well. Please encourage your coauthors to verify their ORCID iD within the submission system before final acceptance, as unverified ORCID iDs will not appear in the published article. *Only*  the individual author can complete the verification step; PLOS staff *cannot*  verify ORCID iDs on behalf of authors.

We look forward to receiving your revised manuscript.

Kind regards,

Georgios Labiris, MD, PhD

Academic Editor

PLOS One

Journal Requirements:

If the reviewer comments include a recommendation to cite specific previously published works, please review and evaluate these publications to determine whether they are relevant and should be cited. There is no requirement to cite these works unless the editor has indicated otherwise.

[Note: HTML markup is below. Please do not edit.]

Reviewers' comments:

Reviewer's Responses to Questions

**Comments to the Author**

Reviewer #1: (No Response)

Reviewer #2: All comments have been addressed

**********

2. Is the manuscript technically sound, and do the data support the conclusions?

Reviewer #1: Yes

Reviewer #2: Partly

**********

3. Has the statistical analysis been performed appropriately and rigorously?

Reviewer #1: Yes

Reviewer #2: I Don't Know

**********

4. Have the authors made all data underlying the findings in their manuscript fully available?

The PLOS Data policy

Reviewer #1: No

Reviewer #2: Yes

**********

5. Is the manuscript presented in an intelligible fashion and written in standard English?

Reviewer #1: Yes

Reviewer #2: Yes

**********

Reviewer #1: The authors have made substantial revisions to the manuscript, which is now more easily comprehendable.

The main general comment is that the quality of all figures is far below standard, thus, many of them are hardly readable. They should be reproduced with higher dpi.

Ideally they should be included in the relevant body of the text, rather than put at the end of the whole document.

I appreciate the description of the steps. Just few clarifications:

Line 140: Step C: is the Gaussian 1D (for each A-scan), or 2D ? If the latter case, is the σ same for both dimensions ? (are the pixels square) ?

Step E. Are the two margins (50μm and 30μm) set manually ?

Step F: The variance of the 1st and 2nd PCA component are set to 94% and 4%, however there seems to be no reason for this to hold in the general case.

Step G: Is the filtering performed in 1D ?

Step H: is the 1.5mm-width window placed in a overlapping manner, or is it applied 4 times to cover the 6mm width ?

Fig.3(a) needs to be explained in the text, because the authors draw important conclusions from it, about the quality of the classifier. The shape is not self-explanatory, the Y axis label is still confusing (does not remind of any probability density function) and the different colors that seem to contain are not visible. Its title is also too general.

Line 341-342 needs rewriting

In Fig. 5b the relevant probability spans [-1,1], whereas in (a) the range is [0,1]

Reviewer #2: I would like to thank the authors for a thorough review of the reviewers’ comments, and a major re-write of the manuscript in order to address them. The manuscript is a lot clearer throughout and the Introduction helps establish the problem motivation well.

After another review of the manuscript, I have some concerns regarding possible data leakage and the results being close to perfect, which is hardly seen in the real world. I would appreciate it if the authors could address the following minor and major comments:

1. Line 69: Sentence structure

2. Line 70: *Additionally*

3. Line 70, 72: *deep-learning*

4. Line 152: Can the authors include a summary of how their normalized cross-correlation method enables a second re-alignment?

5. Line 154: *alignment*

6. Line 184: *mean-centered*

7. Line 208: The statement indicates that the steps A-G would be applied differently to healthy and unhealthy corneas. This could introduce data leakage, and data leakage can potentially nullify later analyses that rely on these steps. Can the authors include in the manuscript how they ensured that that no data leakage occurred during their analyses?

8. Line 301: symbol *alpha*

9. Line 340: Sentence structure

10. Line 382: Can the authors provide data on how many patients were the images collected from, and how many images belonged to the same patient?

11. Line 434: Can the authors clarify whether “unknown” images are a different image dataset than the “FECD” images described previously?

12. Line 433-438: While using SVM to create a reference distribution using single label data can work in some scenarios, it’s not a widely used method, compared to let’s say, Gaussian Discriminative Analysis. As such, this section does not seem to contribute critical information and I would suggest removing it and only focusing on the two-label training.

13. Line 452: *training*

14. Line 460: Accuracy is not a valid metric for imbalanced datasets, so I would suggest removing it.

15. Line 460: Which RF model out of the 5 cross-validated models was used to evaluate the test set?

16. Line 462: I am concerned about the extremely high ROC and predictive power of the model. The results are “too good to be true” and indicate that this is essentially a trivial problem. Since the authors have previously stated that it is not easy to use the images to detect FECD and haze, while at the same time, a random forest model can achieve an ROC of 99.8%, there appears to be a mismatch. I would encourage the authors to carefully review their data generation process to identify and eliminate any “hidden” sources of data leakage.

17. Fig 3a: I have not previously come across this plot format for classification results, and therefore am not able to understand it. It will be helpful to explain the plot in more detail for interpretability.

18. Paragraph 500: It’s unclear how the probability associates with real patient outcomes in the longitudinal study. There does not appear to be any medical diagnosis to establish “ground truth”, which can be compared with the model predictions.

19. Paragraph 524: Since a primary contribution of the manuscript is “interpretability” of the models, I would suggest improving this section by expanding how the Shap values relate to the attributes of the disease, and whether the high importance features align with the expectations of the medical professionals.

20. All figures: Can the authors ensure that the figures in the final publication are high quality as the ones in the manuscript are very low-resolution and difficult to read, though it may be an artifact of journal post-processing?

**********

what does this mean?). If published, this will include your full peer review and any attached files.

If you choose “no”, your identity will remain anonymous but your review may still be made public.

**Do you want your identity to be public for this peer review?**  For information about this choice, including consent withdrawal, please see our Privacy Policy

Reviewer #1: No

Reviewer #2: No

**********

[NOTE: If reviewer comments were submitted as an attachment file, they will be attached to this email and accessible via the submission site. Please log into your account, locate the manuscript record, and check for the action link "View Attachments". If this link does not appear, there are no attachment files.]

To ensure your figures meet our technical requirements, please review our figure guidelines: https://journals.plos.org/plosone/s/figures

You may also use PLOS’s free figure tool, NAAS, to help you prepare publication quality figures: https://journals.plos.org/plosone/s/figures#loc-tools-for-figure-preparation.

NAAS will assess whether your figures meet our technical requirements by comparing each figure against our figure specifications.

# Author comment: Author response to Decision Letter 2

**DOI:** 10.1371/journal.pone.0328320.r004

16 Jul 2026

We would like to thank the reviewer for their detailed and informative comments. We addressed all comments as listed below:

Reviewer #1:

1. Comment 1 reviewer 1:The main general comment is that the quality of all figures is far below standard, thus, many of them are hardly readable. They should be reproduced with higher dpi. Ideally they should be included in the relevant body of the text, rather than put at the end of the whole document.

o Answer 1 (to reviewer 1): We fully agree on the importance of figure quality. The apparent low resolution is an artefact of the journal’s compilation workflow: following the editorial policy, the figures are not embedded in the manuscript file but are uploaded as individual high-resolution TIFF files (>600 dpi), which the editorial system inserts automatically at full resolution in the reviewer PDF. In the present resubmission, per the editorial office’s request, the figures have accordingly been removed from within the manuscript file, leaving only the captions in place, and are provided as separate TIFF image files. We remain happy to adjust figure placement or format according to the journal’s production requirements.

2. Comment 2 reviewer 1:Line 140: Step C: is the Gaussian 1D (for each A-scan), or 2D ? If the latter case, is the σ same for both dimensions ? (are the pixels square) ?

o Answer 2 (to reviewer 1): 2D Gaussian is performed; lateral/axial pixel size varies from 1 to 2 depending on the recording mode of the device (Line, Pachy, Cross, and Pachywide) images; axial pixel size is consistent across modes (4.3um), all modes are considered laterally in a physical distance coordinate system (in mm). To clarify this, we added line 118 (“Standardisation of clinical images” first paragraph): “Images have the same axial pixel size of 4.3um, lateral pixel size varies between 5.85um to 8.51um depending on device recording mode. Images are considered on respective individual coordinate systems.”; We also specified the acquisition mode and its repartition in “Databases and experimental design.”; Added: “2D Gaussian”

3. Comment 3 reviewer 1:Step E. Are the two margins (50μm and 30μm) set manually ?

o Answer 3 (to reviewer 1): Automatically validated by the user. Edited l174: “Automatically excluded”

4. Comment 4 reviewer 1:Step F: The variance of the 1st and 2nd PCA component are set to 94% and 4%, however there seems to be no reason for this to hold in the general case.

o Answer 4 (to reviewer 1): The 94% and 4% values reported for PC1 and PC2 are not preset parameters, but empirically observed values computed by running the PCA on each individual image of the normative dataset (n = 83 normal corneas), following flattening and segmentation of the stroma into 20 equal-thickness sub-layers. The method was developed and iteratively refined on a larger set of normal corneal SD-OCT images prior to this study, providing empirical confidence in the stability of the component interpretation across normal corneas acquired with the same device and protocol. Component selection is not based on these specific percentages, but on the physical interpretation of each component – which is grounded in the known physics of the SD-OCT corneal signal. In this strongly constrained data structure (same device, same protocol, homogeneous normal stroma), PC1 captures the dominant depth-dependent attenuation trend, and PC2 captures the first structured lateral deviation from it, corresponding to the posterior stromal artifact. This interpretation was validated consistently across all images in the dataset. Indirect validation is provided by the fitting results: after correction, stromal attenuation profiles are well described by a mono-exponential decay model (Birge ratio Br < 10 for all normal corneas, IDR: 1.8–4.1), as expected for homogeneous tissue. This would not hold if the PCA-based correction introduced systematic distortions in the depth-attenuation profile. We acknowledge that individual variability around the reported variance values was not documented – these should be understood as representative values observed across the normative dataset, not as fixed algorithmic thresholds.

o We therefore propose the edit by changing the sentence “A first PCA on the full matrix identifies two dominant components: PC1 (94% of variance), capturing the overall depth-dependent attenuation and the curvature-driven lateral intensity decrease, and PC2 (4% of variance), isolating the structured lateral signature of the posterior artefact.” to “A first PCA on the full matrix identifies two dominant components(PC1 and PC2). Across a normative dataset, PC1 and PC2 accounted for approximately 94% and 4% of the variance, respectively. They respectively capture the overall depth-dependent attenuation and the curvature-driven lateral intensity decrease for PC1, and the structured lateral signature of the posterior artefact for PC2 as reported in […].”

5. Comment 5 reviewer 1:Step G: Is the filtering performed in 1D ?

o Answer 5 (to reviewer 1): We are smoothing a curve corresponding to the anterior surface intensity signal. To clarify this: edited l198 to “The corrected image is normalised by the smoothed anterior surface intensity signal (smoothing the detected curve using second-order Savitzky-Golay filter, sliding window: 115 pixels) to compensate for the peripheral intensity loss associated with corneal curvature geometry.”

6. Comment 6 reviewer 1: Step H: is the 1.5mm-width window placed in a overlapping manner, or is it applied 4 times to cover the 6mm width ?

o Answer 6 (to reviewer 1): The 1.5mm-width window is in indeed applied in an overlapping manner (“moving window”), to clarify this we added a footnote for “sliding window” : “sliding window method corresponds to incrementally (pixel-wise) slide a working window, it can mathematically be seen as a convolution by a mean operator with lateral dimension 1.5mm.”

7. Comment 7 reviewer 1: Fig.3(a) needs to be explained in the text, because the authors draw important conclusions from it, about the quality of the classifier. The shape is not self-explanatory, the Y axis label is still confusing (does not remind of any probability density function) and the different colors that seem to contain are not visible. Its title is also too general.

o Answer 7 (to reviewer 1): the Y-axis often represents kernel density estimate (KDE) width, sometimes called “density” or “Probability density”. We propose to edit panel a legend from : “distribution of predicted fibrosis probabilities ($p_{\mathrm{FECD}}$) (Healthy mean predicted probability 10.0\% std: 13.2\%; Fuchs' Dystrophy mean predicted probability: 90.9\% std: 14.2\%; Haze mean predicted probability 96.0\% std: 4.7\%).” To “Distribution of predicted FECD probabilities ($p_{\mathrm{FECD}}$) for healthy corneas, FECD, and visual-haze images in the independent test set. The horizontal axis corresponds to the model-estimated probability of FECD represented by the violin curve. Healthy samples cluster near 0, whereas FECD and visual-haze samples cluster near 1 (healthy mean predicted probability 10.0\% std: 13.2\%; Fuchs' Dystrophy mean predicted probability: 90.9\% std: 14.2\%; Haze mean predicted probability 96.0\% std: 4.7\%).”

o Additionally in the text we edited “reveals a clear separation between the control and pathological groups” to “reveals a clear separation in the x-axis (FECD probability) between the control and pathological groups (y-axis being the model-estimated probability of FECD represented by the violin curve for data distribution visualization).”

o We renamed the y-axis to “Kernel density estimate (a.u.)” for clarity

8. Comment 8 reviewer 1: Line 341-342 needs rewriting

o Answer 8 (to reviewer 1): Text was: “In addition to user quality control of the automatic detection of the artefact removal and these landmarks via the graphical user interface, the pipeline incorporates several complementary mechanisms to maintain robustness when these landmarks are difficult to detect due to elevated noise or pathological morphology”

o Proposed version: “In addition to user-driven quality control via the graphical user interface for validating artefact removal and landmark detection, the pipeline integrates complementary mechanisms to preserve robustness when landmark identification is compromised by high noise levels or pathological morphologies:”

9. Comment 9 reviewer 1: In Fig. 5b the relevant probability spans [-1,1], whereas in (a) the range is [0,1]

o Answer 9 (to reviewer 1): Indeed, right panel represents the value relative to the pre-surgery value, as left panel span initial values from 0 to 1, relatively to initial value it can span from -1 to 1. In the figure caption “Predicted probabilities pFECD (left) and relative to initial pFECD probability (right) of FECD classification in Pre/Post-surgery follow-up for 9 patients.”

o It was also precised line 510 “We found it insightful to represent this follow-up relatively to the initial value of the probability and therefore represent potential improvement or degradation of such quantifier” and line 512 “Comparing Fig 5a and Fig 5b, we can observe that patients with low initial pFECD values tend to maintain high probabilities over time, whereas patients with higher initial pFECD values show a more pronounced decrease, suggesting a potential correlation between the initial severity of fibrosis and the trajectory of recovery.”

Reviewer #2:

1. Comment 1 reviewer 2: Line 69: Sentence structure

o Answer 1 (to reviewer 2): Sentence was : “It is also repported as a competitive compare to deep learning on tabular data” we edit it to “It has also been reported to be competitive with deep learning methods on tabular data”

2. Comment 2 reviewer 2: Line 70: *Additionally*:

o Answer 2 (to reviewer 2): corrected thank you

3. Comment 3 reviewer 2: Line 70, 72: *deep-learning*.

o Answer 3 (to reviewer 2): corrected thank you

4. Comment 4 reviewer 2: Line 152: Can the authors include a summary of how their normalized cross-correlation method enables a second re-alignment?

o Answer 4 (to reviewer 2): Yes, we propose to complete this sentence by: “This second alignment is performed by normalised cross-correlation [17]. Each A-scan is compared to the central A-scan serving as reference: the cross-correlation between the two signals exhibits a peak corresponding to their relative axial offset. Parabolic interpolation around this peak is then applied to achieve sub-pixel estimation of the shift, which is subsequently used to align the A-scans, ensuring sub-pixel co-registration along their first interface.”

5. Comment 5 reviewer 2: Line 154: *alignment*:

o Answer 5 (to reviewer 2): corrected thank you

6. Comment 6 reviewer 2: Line 184: *mean-centered*:

o Answer 6 (to reviewer 2): corrected thank you

7. Comment 7 reviewer 2: Line 208: The statement indicates that the steps A-G would be applied differently to healthy and unhealthy corneas. This could introduce data leakage, and data leakage can potentially nullify later analyses that rely on these steps. Can the authors include in the manuscript how they ensured that that no data leakage occurred during their analyses?

o Answer 7 (to reviewer 2): The sentence is : “The window width of 1.5 mm was selected as a compromise between noise reduction and spatial resolution, on healthy corneas where steps A–G steps was performed automatically without need for manual correction, we observed that the depth-intensity profiles extracted was constant across the 6 mm region of interest for this window width.”

o We believe the confusion comes from “The window width of 1.5 mm was selected […] on healthy corneas”, we didn’t mean to insinuate there was a difference on the processing, and there is in fact no difference in this process, all images a processed similarly. The sentence here missed a “break” : The window width of 1.5 mm was selected as a compromise […]. On healthy corneas”,

o We can reformulate for the sake of clarity: “The window width of 1.5 mm was selected as a compromise between noise reduction and spatial resolution. This parameter was determined a priori and applied uniformly to all samples. In healthy corneas, where steps A–G were performed fully automatically without manual correction, the resulting depth–intensity profiles were observed to be consistent across the 6 mm region of interest for this window width.”

o Image processing and feature extraction were conducted after preprocessing using the same procedure for all samples.

8. Comment 8 reviewer 2: Line 301: symbol *alpha*

o Answer 8 (to reviewer 2): corrected thank you

9. Comment 9 reviewer 2: Line 340: Sentence structure:

o Answer 9 (to reviewer 2): changing sentence “First images are acquired by professionals trained in OCT acquisition and are match with the clinical quality standards” to “images are acquired by professionals trained in OCT acquisition and meet established clinical quality standards”

10. Comment 10 reviewer 2: Line 382: Can the authors provide data on how many patients were the images collected from, and how many images belonged to the same patient?

o Answer 10 (to reviewer 2): Thank you for this comment; table 1 edited, we also added a column for the acquisition mode and a detail on pixel size, as it was also asked in another comment.

11. Comment 11 reviewer 2: Line 434: Can the authors clarify whether “unknown” images are a different image dataset than the “FECD” images described previously?

o Answer 11 In this approach, during training there is no other image than healthy images: unknown images mean image the have never be seen including potential FECD images. Considering next reviewer comment we agreed on removing this part.

12. Comment 12 reviewer 2: Line 433-438: While using SVM to create a reference distribution using single label data can work in some scenarios, it’s not a widely used method, compared to let’s say, Gaussian Discriminative Analysis. As such, this section does not seem to contribute critical information and I would suggest removing it and only focusing on the two-label training.

o Answer 12 (to reviewer 2): We agreed on removing this part.

13. Comment 13 reviewer 2: Line 452: *training*:

o Answer 13 (to reviewer 2): corrected thank you

14. Comment 14 reviewer 2: Line 460: Accuracy is not a valid metric for imbalanced datasets, so I would suggest removing it.

o Answer 14 (to reviewer 2): we agreed

15. Comment 15 reviewer 2: Line 460: Which RF model out of the 5 cross-validated models was used to evaluate the test set?

o Answer 15 (to reviewer 2): Cross-validation was used on the training set to compare candidate models and estimate their performance. The selected model (Random Forest) was then re-instantiated and retrained on the full training dataset using the chosen hyperparameters. This final model was subsequently evaluated on the independent test set.

o We propose the clarification: “[…] Python [25]. Model selection was performed using 5-fold stratified cross-validation on the training set, based on the weighted F1-score, to compare candidate classifiers. The random forest model consistently achieved the best performance and was therefore selected. Following this selection, the final model was re-instantiated and retrained on the full training dataset using the chosen hyperparameters. On the held-out test set […]”

16. Comment 16 reviewer 2: Line 462: I am concerned about the extremely high ROC and predictive power of the model. The results are “too good to be true” and indicate that this is essentially a trivial problem. Since the authors have previously stated that it is not easy to use the images to detect FECD and haze, while at the same time, a random forest model can achieve an ROC of 99.8%, there appears to be a mismatch. I would encourage the authors to carefully review their data generation process to identify and eliminate any “hidden” sources of data leakage.

o Answer 16 (to reviewer 2): We understand the reviewer's comment, and carefully investigated for an eventual leakage, however images are processed similarly for each condition, devices, and practitioners are shared for each condition. We also want to highlight that we trained and tested the model on a set of images and further tested it as presented in the manuscript on repeatability analysis for both condition-varying practitioners. In our introduction, we emphasized the need for quantitative, objective, and reproducible, not hard we focus firstly on the need for objective, quantitative and reproducible analysis. It is now accepted that machine learning can accurately reproduce human judgement in many cases when done carefully, as we tried to do here. This is still a proof of principle, however, and evaluation on larger, multicentric datasets acquired by diverse acquisition devices, protocols, and practitioners may raise additional challenges

17. Comment 17 reviewer 2: Fig 3a: I have not previously come across this plot format for classification results, and therefore am not able to understand it. It will be helpful to explain the plot in more detail for interpretability.

o Answer 17 (to reviewer 2): the Y-axis often represents kernel density estimate (KDE) width, sometimes called “density” or “Probability density”. We propose to edit panel a legend from : “distribution of predicted fibrosis probabilities ($p_{\mathrm{FECD}}$) (Healthy mean predicted probability 10.0\% std: 13.2\%; Fuchs' Dystrophy mean predicted probability: 90.9\% std: 14.2\%; Haze mean predicted probability 96.0\% std: 4.7\%).” To “Distribution of predicted FECD probabilities ($p_{\mathrm{FECD}}$) for healthy corneas, FECD, and visual-haze images in the independent test set. The horizontal axis corresponds to the model-estimated probability of FECD represented by the violin curve. Healthy samples cluster near 0, whereas FECD and visual-haze samples cluster near 1 (healthy mean predicted probability 10.0\% std: 13.2\%; Fuchs' Dystrophy mean predicted probability: 90.9\% std: 14.2\%; Haze mean predicted probability 96.0\% std: 4.7\%).”

o Additionally in the text we edited “reveals a clear separation between the control and pathological groups” to “reveals a clear separation in the x-axis (FECD probability) between the control and pathological groups (y-axis being the model-estimated probability of FECD represented by the violin curve for data distribution visualization).”

o We renamed the y-axis to “Kernel density estimate (a.u.)” for clarity

18. Comment 18 reviewer 2: Paragraph 500: It’s unclear how the probability associates with real patient outcomes in the longitudinal study. There does not appear to be any medical diagnosis to establish “ground truth”, which can be compared with the model predictions.

o Answer 18 (to reviewer 2): This is an exploratory analysis, investigating further uses of the proposed model. It will need to be strengthened by a proper longitudinal study where a clinical baseline would be constructed for a proper clinical evaluation. As we are exploring the potential application of the automatic and reproducible quantification we are proposing.

19. Comment 19 reviewer 2: Paragraph 524: Since a primary contribution of the manuscript is “interpretability” of the models, I would suggest improving this section by expanding how the Shap values relate to the attributes of the disease and whether the high-importance features align with the expectations of the medical professionals.

o Answer 19 (to reviewer 2): The nine quantifiers have been build to probe the specific features described the 3 morphological feature described in “Morphological parameters of subepithelial fibrosis”. We propose to edit the l528 : « higher values of peak width, sigma, area ratio, and depth; and lower values of beta (reduced stromal intensity signal decay) are associated with higher SHAP values” by adding: “Those parameters relating by construction to the 3 morphological parameters of subepithelial fibrosis : sub-epithelial hyper reflectivity (link to area ratio), blurred epithelium/stroma interface (link to peak width, sigma and depth), altered reflectivity depth profile, stromal transparency (link to beta).”

20. Comment 20 reviewer 2: All figures: Can the authors ensure that the figures in the final publication are high quality as the ones in the manuscript are very low-resolution and difficult to read, though it may be an artifact of journal post-processing?

o Answer 20 (to reviewer 2): Indeed, the pixelated appearance is an artefact of the journal’s post-processing: following the editorial policy, images are removed from the manuscript file and uploaded individually as high-resolution TIFF files (>600 dpi), which the editorial system inserts automatically at full resolution in the compiled PDF. In this resubmission, per the editorial office’s request, the figures have been removed from within the manuscript file (captions retained) and are provided as separate TIFF image files, ensuring that the versions appearing in the final publication are of full quality.

Attachment Submitted filename: Responses_To_Reviewers_3.docx

# Peer review: Decision Letter 2

**DOI:** 10.1371/journal.pone.0328320.r005

**Authors:** Helen Howard

27 Sep 2026

Machine learning quantitative OCT derived fibrosis indicators for corneal haze evaluation

PONE-D-25-32169R2

Dear Dr. Soubeiran,

We’re pleased to inform you that your manuscript has been judged scientifically suitable for publication and will be formally accepted for publication once it meets all outstanding technical requirements.

Within one week, you’ll receive an e-mail detailing the required amendments. When these have been addressed, you’ll receive a formal acceptance letter and your manuscript will be scheduled for publication.

An invoice will be generated when your article is formally accepted. Please note, if your institution has a publishing partnership with PLOS and your article meets the relevant criteria, all or part of your publication costs will be covered. Please make sure your user information is up-to-date by logging into Editorial Manager at Editorial Manager® and clicking the ‘Update My Information' link at the top of the page. For questions related to billing, please contact billing support.

If your institution or institutions have a press office, please notify them about your upcoming paper to help maximize its impact. If they’ll be preparing press materials, please inform our press team as soon as possible -- no later than 48 hours after receiving the formal acceptance. Your manuscript will remain under strict press embargo until 2 pm Eastern Time on the date of publication. For more information, please contact onepress@plos.org.

Kind regards,

Helen Howard

Staff Editor

PLOS One

Additional Editor Comments (optional):

Reviewers' comments:

Reviewer's Responses to Questions

**Comments to the Author**

Reviewer #2: All comments have been addressed

**********

2. Is the manuscript technically sound, and do the data support the conclusions?

Reviewer #2: Yes

**********

3. Has the statistical analysis been performed appropriately and rigorously?

Reviewer #2: Yes

**********

4. Have the authors made all data underlying the findings in their manuscript fully available?

The PLOS Data policy

Reviewer #2: No

**********

5. Is the manuscript presented in an intelligible fashion and written in standard English?

Reviewer #2: Yes

**********

Reviewer #2: (No Response)

**********

what does this mean?). If published, this will include your full peer review and any attached files.

If you choose “no”, your identity will remain anonymous but your review may still be made public.

**Do you want your identity to be public for this peer review?**  For information about this choice, including consent withdrawal, please see our Privacy Policy

Reviewer #2: No

**********

# Editor report: Acceptance letter

**DOI:** 10.1371/journal.pone.0328320.r006

**Authors:** Helen Howard

PONE-D-25-32169R2

PLOS One

Dear Dr. Soubeiran,

I'm pleased to inform you that your manuscript has been deemed suitable for publication in PLOS One. Congratulations! Your manuscript is now being handed over to our production team.

At this stage, our production department will prepare your paper for publication. This includes ensuring the following:

* All references, tables, and figures are properly cited

* All relevant supporting information is included in the manuscript submission,

* There are no issues that prevent the paper from being properly typeset

You will receive further instructions from the production team, including instructions on how to review your proof when it is ready. Please keep in mind that we are working through a large volume of accepted articles, so please give us a few days to review your paper and let you know the next and final steps.

Lastly, if your institution or institutions have a press office, please let them know about your upcoming paper now to help maximize its impact. If they'll be preparing press materials, please inform our press team within the next 48 hours. Your manuscript will remain under strict press embargo until 2 pm Eastern Time on the date of publication. For more information, please contact onepress@plos.org.

You will receive an invoice from PLOS for your publication fee after your manuscript has reached the completed accept phase. If you receive an email requesting payment before acceptance or for any other service, this may be a phishing scheme. Learn how to identify phishing emails and protect your accounts at https://explore.plos.org/phishing.

If we can help with anything else, please email us at customercare@plos.org.

Thank you for submitting your work to PLOS One and supporting open access.

Kind regards,

PLOS One Editorial Office Staff

on behalf of

Dr Helen Howard

Staff Editor

PLOS One
